# Novel roles of DC-SIGNR in colon cancer cell adhesion, migration, invasion, and liver metastasis

**DOI:** 10.1186/s13045-016-0383-x

**Published:** 2017-01-21

**Authors:** Heya Na, Xiaoli Liu, Xiaomeng Li, Xinsheng Zhang, Yu Wang, Zhaohui Wang, Menglang Yuan, Yu Zhang, Shuangyi Ren, Yunfei Zuo

**Affiliations:** 1 Department of Clinical Biochemistry, College of Laboratory Diagnostic Medicine, Dalian Medical University, Dalian, 116044 China; 2 Department of General Surgery, The Second Affiliated Hospital of Dalian Medical University, Dalian, 116023 China; 3 Department of Clinical Laboratory, The Second Affiliated Hospital of Dalian Medical University, Dalian, 116023 China

**Keywords:** DC-SIGNR, Colon cancer liver metastasis, Metallothioneins

## Abstract

**Background:**

Tumor metastasis is an essential cause of the poor prognosis of colon cancer. DC-SIGNR is a C-type lectin that is frequently found on human liver sinusoidal endothelial cells. LSECtin, which is a homologue of DC-SIGNR, has been demonstrated to participate in colon cancer liver metastasis. Due to the similarities in the expression pattern and structure of the two proteins, we speculated that DC-SIGNR could also be involved in this process.

**Methods:**

Colon cancer cells were treated with the DC-SIGNR protein or control IgG, after which cell migration, invasion, and morphology were assayed. Xenograft mouse models were used to determine the role of DC-SIGNR in colon cancer liver metastasis in vivo. In addition, a human gene expression array was used to detect differential gene expression in colon cancer cells stimulated with the DC-SIGNR protein. The serum level of DC-SIGNR was examined in colon cancer patients by ELISA, and the significance of DC-SIGNR was determined.

**Results:**

In our research, we investigated whether DC-SIGNR promotes colon cancer cell adhesion, migration, and invasion. Knocking down mouse DC-SIGNR decreased the liver metastatic potency of colon cancer cells and increased survival time. Expressing human DC-SIGNR enhanced colon cancer liver metastasis. Furthermore, DC-SIGNR conferred metastatic capability on cancer cells by upregulating various metallothionein isoforms. To validate the above results, we also found that the serum DC-SIGNR level was statistically higher in colon cancer patients with liver metastasis compared with those without metastasis.

**Conclusions:**

These results imply that DC-SIGNR may promote colon carcinoma hepatic metastasis and could serve as a promising therapeutic target for anticancer treatment.

**Electronic supplementary material:**

The online version of this article (doi:10.1186/s13045-016-0383-x) contains supplementary material, which is available to authorized users.

## Background

Tumor metastasis, the main feature of malignant tumors, is often considered to be the most lethal property of cancer cells [1]. The process of tumor metastasis is complex, continuous, and multifactorial. Tissue-specific interactions play a critical role in the initiation of tumor metastasis. This process is regulated by specific tissue cells as well as by cancer cells [2, 3]. From the perspective of the organ microenvironment, both the vessel diameter of capillary walls and the structural composition of blood vessels can affect tumour cell infiltration [4, 5]. The capillaries of the liver and lymph nodes are found to be lined with fenestrated endothelial cells and are thus more permissive to the recruitment of tumour cells. Furthermore, some specific ligands on the endothelial cells are also involved in tumor metastasis. At the same time, tumor cells can optimize their motility depending on the environment of the surrounding tissues and regulate the expression of specific receptors on the cell surface, which synergistically facilitate organ-specific metastasis [6].

DC-SIGNR, dendritic cell-specific intercellular adhesion molecule-grabbing nonintegrin-related protein, is a C-type II integral membrane protein. It is predominantly found on the sinusoidal endothelial cells in the liver and lymph nodes as well as in the placental endothelium [7]. Generally, the importance of DC-SIGNR is due to its role as a cell-adhesion receptor that has high affinity for ICAM-2, ICAM-3, Lewis^a^, Lewis^b^, and Lewis^y^ [8]. DC-SIGNR is also involved in the innate immune system and recognizes diverse pathogens, ranging from viruses to parasites, that have a tremendous impact on public health. It has been reported to play an important role in various pathophysiological conditions, such as hepatitis C, HIV, and severe acute respiratory syndrome [9–11]. LSECtin belongs to a subfamily that includes DC-SIGNR, DC-SIGN, and CD23 and is also found on the liver sinusoidal endothelial cells. It has been demonstrated that LSECtin is a crucial regulator of colon cancer liver metastasis [12]. The serum LSECtin level in patients with colon cancer liver metastasis was higher than that in patients without metastasis, and LSECtin increased the expression of c-Met in colon cancer cells. Recently, our laboratory has reported that the level of serum DC-SIGNR in colon cancer patients was higher than in healthy volunteers [13]. DC-SIGNR may be a significant biomarker that could be used for the diagnosis of early stage colon cancer. Furthermore, many other C-type lectins have also been demonstrated to be involved in tumour metastasis. Christopher R. Parish et al. found that endothelial-derived P-selectin was as important as platelet-derived P-selectin in aggravating lung metastases and participated in liver metastasis [14]. Sayeda Yasmin-Karim emphasized that the interaction of E-selectin and the E-selectin ligand mediated the rolling and adhesion of prostate cancer cells on the endothelial surface through the RAS-ERK-MAP kinase pathway [15]. The mannose receptor promoted colon carcinoma cell interaction with the liver sinusoidal endothelium and contributed to colon cancer hepatic metastasis, which was induced by interleukin-1 [16]. Therefore, we hypothesized that DC-SIGNR could also be associated with human colon cancer progression.

Metallothioneins are a group of evolutionarily conserved, low molecular weight, cysteine-rich proteins. The metallothionein genes are located on chromosome 16 (16q12-22) in humans. There are four primary metallothionein isoforms: MT1, MT2, MT3, and MT4. Whereas MT1 and MT2 are commonly expressed, MT3 seems to be restricted to the brain, and MT4 is specifically found in certain squamous epithelial tissues. Furthermore, there are eight functional paralogs of MT1 (MT1A, MT1B, MT1E, MT1F, MT1G, MT1H, MT1M, and MT1X) [17]. Metallothioneins, as redox-active proteins, have many functions, including zinc homeostasis, heavy metal detoxification and metal transfer [18]. They can facilitate the chemoresistance to some anti-cancer drugs [19, 20]. In recent years, metallothioneins have been shown to be related to tumor stage, therapy resistance, poor prognosis, and survival in many cancers [21].

In our study, we aimed to gain insights into the important biological effect of DC-SIGNR on colon cancer. We found that colon cancer patients with liver metastasis showed higher levels of DC-SIGNR compared with patients without metastasis. In addition, DC-SIGNR expression could be induced on colon cancer cells through various changes in the tumour microenvironment. Furthermore, DC-SIGNR mediated the upregulation of metallothioneins. Both in vitro and in vivo experiments were performed to elucidate the relevance of DC-SIGNR-mediated tumor metastasis, and the inhibition of DC-SIGNR activity or of downstream DC-SIGNR-regulated events could be a therapeutic intervention for colon cancer formation.

## Methods

### Cell culture and nude mice

Colon cancer cells LoVo, LS174T and HCT-116 were used in this research. Cells were cultured in RPMI 1640 medium supplemented with 10% fetal bovine serum and 100 U/ml penicillin and streptomycin (Beyotime, China). Cells were maintained in a 37 °C incubator with 5% CO_2_ humidified air. Six-to-eight-week old nude mice were purchased from Dalian Medical University (Dalian, China). They were bred and acclimated under specific pathogen-free conditions. All of the animal studies were conducted in accordance with the Institutional Animal Care and Use Committee of Dalian Medical University.

### Patients

A total of 81 patients with colon cancer were enrolled in our study, and all patients were treated in the First or Second Affiliated Hospital of Dalian Medical University. None of the patients were immediate relatives. Clinical information about patients is listed in Additional file 1: Table S2. The control group consisted of 57 healthy volunteers. These clinical materials for research were approved by the Research Ethics Committee of Dalian Medical University.

### Plasmid

DC-SIGNR expression plasmid was produced and depended on cDNA fragment containing the full coding sequence of DC-SIGNR ((nt)39-1184, AF290887). Two shRNA sequences for knockdown of mouse DC-SIGNR were shRNA a: 5′-GCAGACTTCTAAGGCTAAAGG-3′; shRNA b 5′-GCTCCAGA CTACGACCAAATT-3′. The negative control vector contains a nonsense sequence which has no significant homology with the target sequence. It was 5′-GTTCTCCGAACGTGTCACGT-3′. All of the plasmids were designed and synthesized by Shanghai Gene Pharma (Shanghai, China).

### Detection of soluble DC-SIGNR in colon cancer patients

The serum levels of DC-SIGNR were measured using a commercial sandwich enzyme-linked immunosorbent assay method. The wells of 96-well microplates were coated with 100 μl of an anti-DC-SIGNR goat polyclonal antibody (1:1000, Santa Cruz Biotechnology, Inc., USA) at a final concentration of 0.27 μg/ml as a capture antibody and incubated overnight at 4 °C. The plates were washed three times with phosphate buffered saline (PBS) containing 0.05% Tween-20 (PBST, pH 7.4), and the wells were blocked with blocking buffer (5% bovine serum albumin) at 37 °C for 1 h. The plates were washed three times with PBST. Then, 100 μl of diluted rhDC-SIGNR-Fc standard was added to the wells along with the serum from colon cancer patients and healthy controls. As a negative control, 100 μl of PBS was used. Each plate was incubated at 37 °C for 2 h. Subsequently, PBST was used to wash the plates three times, and 100 μl of an anti-DC-SIGNR mouse monoclonal antibody (1:1000, R&D Systems, Inc., USA) diluted to a concentration of 0.5 μg/ml was added to each well. The plates were incubated at 37 °C for 1.5 h. After washing, 100 μl of a peroxidase-conjugated goat anti-mouse antibody (1:1000, ZSGB-BIO, China) was added, and the plates were incubated at 37 °C for 1 h. Finally, the plates were washed three times and incubated with 3, 3′, 5, 5′-tetramethylbenzidine (TMB, TIANGEN BIOTECH CO, LTD.) at 37 °C for 0.5 h, and then 2 mol/L H_2_SO4 was added to stop the reaction. The optical density (OD) was measured at a wavelength of 450 nm in a microplate reader. The quantitative DC-SIGNR concentrations were determined by comparing the optical density values with the standard curve (Additional file 2: Figure S5).

### Cell adhesion and flow cytometry

The colon cancer cell lines LoVo, LS174T and HCT-116 were treated with a recombinant human DC-SIGNR protein or control IgG on ice for 3 h. To detect the interaction between the cells and the protein, the cells were incubated with an anti-DC-SIGNR mouse monoclonal antibody (1:100, R&D Systems, Inc., USA) on ice for 1 h. The cells were gently washed three times with PBS. Then, a fluorescein isothiocyanate-conjugated goat anti-mouse IgG secondary antibody (1:50, ZSGB-BIO, China) was added, and the cells were incubated on ice for another 1 h. The cells were then washed and fixed in 4% paraformaldehyde. The adherent cells were measured the next day using a FACSCalibur instrument (BD Biosciences, USA). For the inhibition assay, the DC-SIGNR protein (5 μg/ml) was pre-incubated with selected ligands, including EDTA, D-mannose, galactose, N-acetylglucosamine, and L-fucose.

### In vitro migration and invasion assays

Cells were treated with the recombinant DC-SIGNR protein or with control IgG. The in vitro migration assay was performed in 24-well plates with Transwell inserts (6.5 mm in diameter, 8-μm pore size, Corning, USA). For the invasion assay, the cells were incubated on the Matrigel-coated membranes of the inserts. The cells were incubated for 24 h, and then the cells on the upper membrane, which had not migrated or invaded, were removed using a cotton swab. Subsequently, the cells on the lower side of the membrane were fixed in methanol for 15 min and stained with 0.5% crystal violet for 30 min. For the wound healing assay, cell monolayers were wounded by a sterile pipet tip when the cells were nearly 100% confluent. The movement of the cells was photographically monitored at 0 and 24 h after the wounding. Images were taken at the indicated time points (Olympus IX71, Japan).

### Colony formation assay

LoVo, LS174T, and HCT-116 colon cancer cells were seeded in six-well plates at a density of 1000 cells per well. The cells were treated with 5 μg/ml DC-SIGNR protein or control IgG. After incubating for 14 days at 37 °C in a humidified 5% CO_2_ atmosphere, visible clones appeared. The cells were washed with PBS and stained with 0.5% crystal violet in methanol for 45 min. Colonies with >50 cells were counted using an optical microscope (Olympus IX71, Japan).

### Injection of plasmid DNA

Naked plasmid DNA was administered to mice in a large volume via tail vein injection. The volume of the solution used corresponded to 8% of the body weight of the mouse, i.e., 1.6–1.8 ml. The injections were completed within 5–7 s.

### Reverse transcription polymerase chain reaction

Total RNA was isolated from liver tissues using TRIzol reagent (TaKaRa Bio, Japan). The human DC-SIGNR transcript was amplified with the following primers: forward primer, 5′- TGTCCAAGGTCCCCAGCTCCC-3′, reverse primer, 5′-GAACTCACCAAATGCAGTCTTCAAATC-3′. The mouse DC-SIGNR primers were: forward primer, 5′-TGGCAGTCTCCAAAACCCCAAATAC-3′, reverse primer, 5′-ACGGCGTCATTCCAGTTCCGC-3′. β-actin was selected as the endogenous control and was amplified using the following primers: forward primer 5′-GGCTGTATTCCCCTCCATCG-3′, reverse primer 5′-CCAGTTGGTAACAATGCCATGT-3′. The PCR amplification products were distinguished on a 1% agarose (Pronadisa, Spain) gel containing 0.5 μg/ml Gold View I and visualized under a UV transilluminator.

### Western blot analysis

Proteins from mouse liver tissues and from LoVo, LS174T and HCT-116 cells were extracted and separated on Tris-glycine gels, and western blot analyses were performed according to standard procedures. The protein extracts were immunoblotted with a mouse DC-SIGNR antibody (1:1000, R&D Systems, Inc., USA), human DC-SIGNR antibody (1:500, Abcam, USA), metallothionein antibody (1:150, Biosynthesis Biotechnology, China), and MMP9 antibody (1:1000, Elabscience Biotechnology, China). A β-actin antibody was used as a control.

### Establishment of the liver metastatic model

Mice were anaesthetized with pentobarbital sodium (Sigma, USA) at 45 mg/kg body weight by intrasplenic injection. A 1-cm incision was made in the left flank, and the spleen was separated and exteriorized. Then, 5 × 10^6^ LoVo, LS174T or HCT-116 cells were injected into the spleen. The spleen was returned to the abdominal cavity, and the wound was sutured. When signs of abdominal distension or locomotive deficit appeared or a tumor was detected by palpation, the mice were killed, and their livers and spleens were harvested.

### Immunohistochemistry analysis

Tissue slides were deparaffinized, rehydrated and boiled in citrate buffer. The tissue sections were incubated with primary Ki67 (1:100, Bioworld Technology, China), CEA (1:100, Bioworld Technology, China), CK20 (1:100, Bioworld Technology, China) and CK7 (1:100, Proteintech, China) antibodies overnight at 4 °C. Then, the sections were incubated with an HRP-conjugated anti-rabbit antibody (1:1000, ZSGB-BIO, China) for 30 min at 37 °C, stained with DAB, and counterstained with haematoxylin.

### Human gene expression array

LoVo cells were incubated with the recombinant DC-SIGNR protein or control IgG for 4 h. Total RNA was isolated and analyzed on an Affymetrix GeneChip PrimeView^TM^ Human Gene Expression Array (Affymetrix, Santa Clara, CA). On this chip, 530,000 probe sets represent more than 20,000 genes, which are completely annotated by the RefSeq RNA database.

### Quantitative real-time PCR validation

Quantitative real-time PCR was used to validate the changes in expression of metallothioneins and MMP9 in colon cancer cells. cDNA was synthesized using a PrimeScript II 1st Strand cDNA Synthesis Kit (TaKaRa Bio, Japan). All PCR reactions were performed at least in triplicate, and the results were analysed on a Thermal Cycler Dice Real-Time System (TaKaRa Bio, Japan) using SYBR Premix Ex TaqTM PCR master mix (TaKaRa Bio, Japan). GAPDH was selected as the endogenous control. The primer pairs used for PCR are presented in Additional file 3: Table S3. The fold change of each gene was calculated using the delta-delta CT method.

### Statistical analysis

The data were presented as the mean ± SD as indicated. Comparisons between two groups were assessed using an unpaired two-tailed *t* test. A one-way ANOVA with Tukey’s Multiple Test were used for comparisons between multiple groups. The non-parametric Mann-Whiney *U* test was employed to analyse the association of DC-SIGNR levels with various clinicopathologic characteristics. The survival analysis was performed using the log-rank (Mantel-Cox) test. For all tests, a *P* value of <0.05 was considered significant. All results were reproduced across triplicate experiments, and the statistical analyses were carried out using GraphPad Prism (GraphPad Software, Inc., USA).

## Results

### Recombinant DC-SIGNR protein adheres to LoVo, LS174T, and HCT-116 cells

**Fig. 1:**
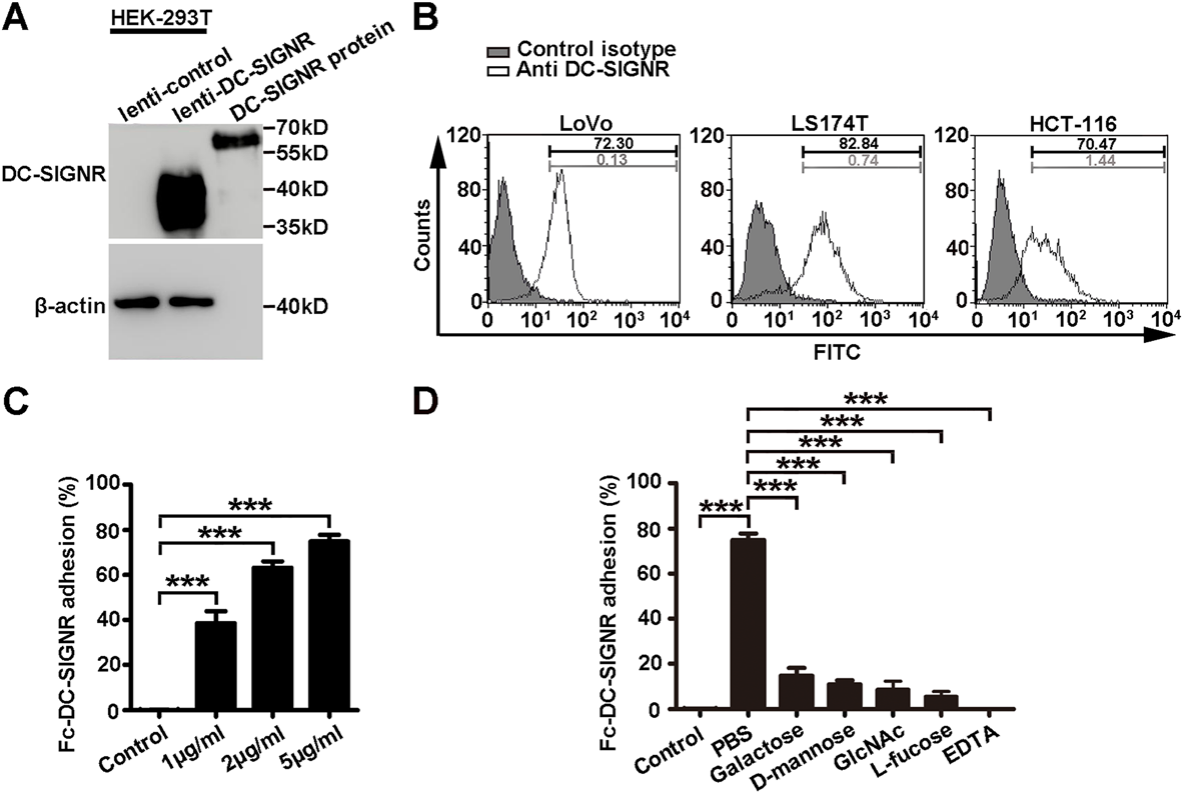
DC-SIGNR regulates colon cancer cell adhesion. **a** The DC-SIGNR protein was detected by Western Blot. **b** LoVo, LS174T, and HCT-116 cells were incubated with the recombinant DC-SIGNR protein on ice for 3 h. The adhesion complexes were detected by an antibody against DC-SIGNR and a FITC-conjugated goat anti-mouse IgG secondary antibody and analysed by flow cytometry. The adhesive ratio of the three colon cancer cell lines was 72.30% for the LoVo cells, 82.84% for the LS174T cells and 70.47% for the HCT-116 cells. **c, d** LoVo cells were treated with different concentrations of the DC-SIGNR protein (1, 2 and 5 μg/ml). For the inhibition assay, the DC-SIGNR (5 μg/ml) protein was pre-incubated with EDTA, galactose, D-mannose, N-acetylglucosamine, or L-fucose. The binding of DC-SIGNR to LoVo cells was dose-dependent, and the interaction could be inhibited by EDTA and monosaccharides. ***P < 0.001. The *error bars* in all graphs represent SD, and each experiment was repeated three times

Because DC-SIGNR acts as an adhesion receptor, we first wondered whether DC-SIGNR was associated with the metastatic potential of colon cancer cells. We examined the capability of the DC-SIGNR protein to bind to colon cancer cells. The DC-SIGNR recombinant protein (R&D Systems, Inc., USA) encodes the extracellular domain (Ser 78-Glu 399) of human DC-SIGNR and is stably expressed in mouse myeloma cell line (derived from NS0 cell, the non-Ig secreting and non-light chain-synthesizing cell line) by Gene engineering technique. By a series of extraction and purification process, the Fc-DC-SIGNR Chimera is generated. It has been used in many applications [13, 22]. We verified the expression of human Fc-DC-SIGNR by Western Blot analysis (Fig. 1a). We used HEK-293T cells infected with a lentivirus expressing DC-SIGNR as a positive control [23]. The expression of DC-SIGNR was detected using a DC-SIGNR primary antibody (1:2000, Abcam, USA) and a peroxidase-conjugated anti-rabbit IgG secondary antibody (1:4000, ZSGB-BIO, China). The predicted molecular weight for the antibody is 45 kDa. In addition, the predicted molecular weight of our recombinant human DC-SIGNR chimera protein is 61.4 kDa, based on its migration on an SDS-PAGE gel. We then treated three colon cancer cell lines, LoVo, LS174T, and HCT-116, with human DC-SIGNR or a mouse IgG isotype control on ice for 3 h. The mouse IgG isotype control was used to block any nonspecific binding sites of the anti-DC-SIGNR mouse primary antibody. The results indicated that the DC-SIGNR protein bound strongly to these three cell types. The respective adhesive ratios were 72.30% for LoVo cells, 82.84% for LS174T cells, and 70.47% for HCT-116 cells (Fig. 1b). Notably, the binding of the DC-SIGNR protein to LoVo cells occurred in a dose-dependent manner (Fig. 1c). DC-SIGNR is a C-type II transmembrane lectin containing a calcium-dependent carbohydrate recognition domain (CRD) and a second site analogous to that identified in mannose-binding protein [24]. In addition, DC-SIGNR selectively binds some monosaccharides in a Ca^2+^-dependent manner, suggesting that the binding sites are analogous to those observed in other C-type lectin CRDs [7, 25]. Therefore, we sought to determine whether DC-SIGNR could recognize ligands on colon cancer cells through calcium- and mannose-dependent binding. The results showed that the binding of DC-SIGNR to colon cancer cells required the presence of Ca^2+^, as this binding was inhibited by the addition of a Ca^2+^ binding chelator (EDTA) (Fig. 1d). The interaction could also be blocked by the addition of some monosaccharides, namely, D-mannose, galactose, N-acetylglucosamine, and L-fucose (Fig. 1d). Thus, these data indicate that the interaction between DC-SIGNR and colon cancer cells may be calcium-dependent and that DC-SIGNR may bind to colon cancer cells through a protein-glycan interaction.

### DC-SIGNR positively regulates colon cancer cell migration and invasion independent of cellular proliferation

**Fig. 2:**
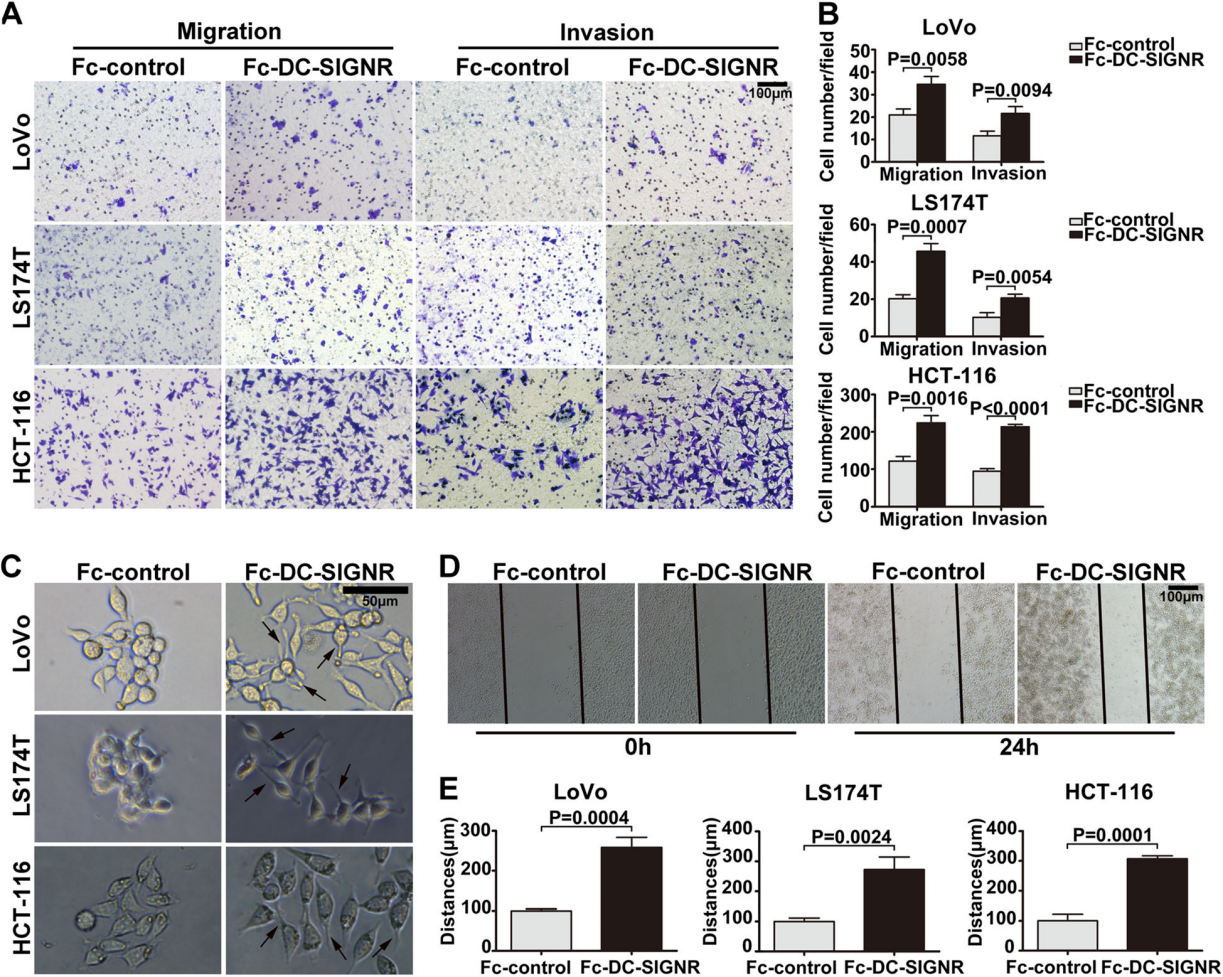
DC-SIGNR promotes colon cancer cell migration and invasion in vitro. **a** Crystal violet staining showed that DC-SIGNR positively promoted the migration and invasion ability of the three colon cancer cell lines. *Scale bars*, 100 μm. **b** The in vitro migration and invasion promoting activities of colon cancer cells treated with the DC-SIGNR protein or control IgG were measured by determining the number of cells that passed through the Transwell membrane. **c** Morphology micrographs of the three types of colon cancer cells treated with the DC-SIGNR protein or control IgG for 24 h. The DC-SIGNR protein induced scattering of the three types of colon cancer cells. Scale bars, 50 μm. **d** Pictures of the wound healing assay in LoVo cells incubated with the DC-SIGNR protein or control IgG at 24 h after the cells were scratched. The LoVo cells treated with the DC-SIGNR protein migrated more quickly than those treated with control IgG. *Scale bars*, 100 μm. **e** The relative cell migrations of the three colon cancer cell lines at 24 h after the cells were scratched are shown in this panel. The *error bars* in all graphs represent SD, and the results were quantified from three experiments

The other two main characteristics of metastatic cancer cells are migration and their ability to degrade physical barriers, such as extracellular matrix components, to invade new tissues. To assess the effect of DC-SIGNR on cell motility and invasion, we used Transwell inserts as described above. We found that the DC-SIGNR protein markedly enhanced the migration of the three types of colon cancer cells and that more cells invaded into the Matrigel^TM^ in the DC-SIGNR protein-treated group than in the untreated group (Fig. 2a, b). To further validate these results, we also observed the cell morphology and performed a wound healing assay. By comparing the morphology of the cells described above under a light microscope, we observed that DC-SIGNR could induce scattering of the colon cancer cells and change the morphology of the cells from epithelial-like to a more elongated cell shape (Fig. 2c). This is typified by a morphologic polarization consisting of the formation of protrusions in the process of cell migration [26, 27]. Additionally, in a wound-healing assay, the cancer cells treated with the DC-SIGNR protein were able to heal the inflicted wound significantly faster than the untreated control cells (Fig. 2d, e). Taken together, these data imply that DC-SIGNR may confer migration and invasion abilities on colon cancer cells.

To confirm whether the results of the in vitro migration and invasion assays were due to differences in cellular proliferation between the three types of colon cancer cells, we measured the cell growth rates using a colony formation assay. No significant difference in the number of colonies was observed between the cells treated with the DC-SIGNR protein and those treated with control IgG (Additional file 4: Figure S1A and B). Taken together, these data imply that DC-SIGNR confers migration and invasion abilities on colon cancer cells without altering the proliferation of the cells.

### Mouse DC-SIGNR knockdown inhibits the liver metastatic capacity of colon cancer cells in vivo

**Fig. 3:**
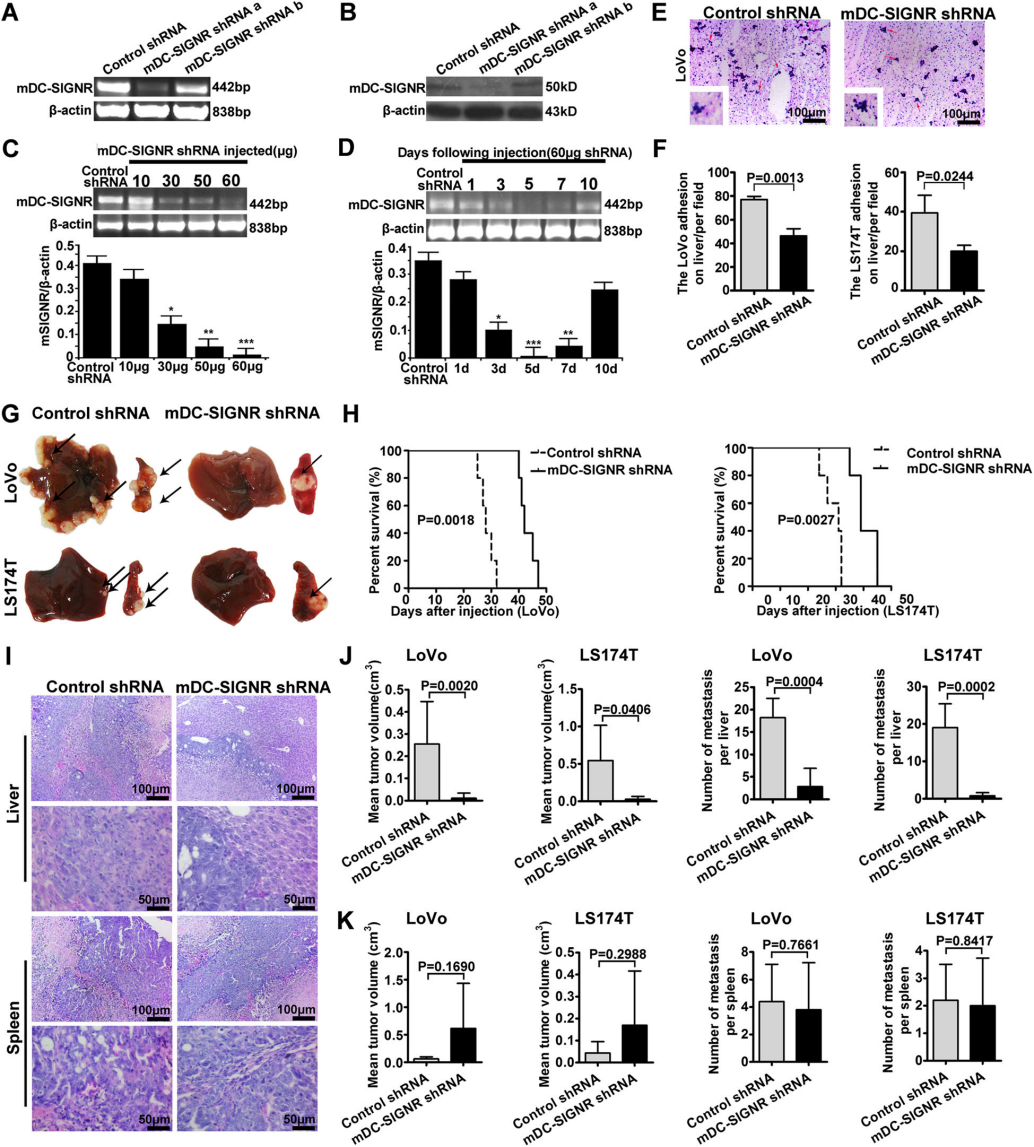
Mouse DC-SIGNR knockdown inhibits colon cancer liver metastasis in vivo. **a, b** The effects of two different mouse DC-SIGNR shRNA constructs on the expression of mouse DC-SIGNR were detected by RT-PCR and Western Blot analysis. Liver tissue from mice injected with a non-targeting shRNA plasmid was used as the positive control. β-actin was used as a loading control. **c** The effect of different doses of the mouse DC-SIGNR shRNA plasmid on the expression of mouse DC-SIGNR was analysed by RT-PCR. The greyscale analysis of the mouse DC-SIGNR bands was normalized to β-actin. **d** RT-PCR analysis was used to analyse the mouse DC-SIGNR expression in the mouse livers at the indicated time points following the injection of 60 μg of the mouse DC-SIGNR shRNA plasmid. The greyscale analysis of the mouse DC-SIGNR bands was normalized to β-actin. ***P < 0.001. **e**, **f** Liver sections were extracted from mice 3 days after injection with the mDC-SIGNR shRNA plasmid or a control shRNA plasmid via tail vein. LoVo cells that had adhered to frozen liver sections were detected by H&E staining. The adherent colon cancer cells are labelled with red arrows. **g** Representative sections of liver tumors (*n* = 5 in each group). The *black arrows* indicate the tumor nodules. Knocking down mouse DC-SIGNR significantly decreased the metastasis of LoVo and LS174T cells to the liver. **h** Comparison of the survival percentage of the mouse DC-SIGNR shRNA group and control shRNA group. The survival percentage of mice in the mouse DC-SIGNR shRNA group was significantly higher than that of the control shRNA group for both of the colon cancer cell lines (*P* = 0.0018 and *P* = 0.0027, respectively). **i** Haematoxylin and eosin (H&E)-stained liver and spleen sections are shown. *Scale bars*, 50 μm and 100 μm, respectively. **j** The mean volume of tumors and the number of metastases in the liver were determined. **k** The mean volume of tumors and the number of metastases in the spleen were determined. *P < 0.05, **P < 0.01, ***P < 0.001. The *error bars* in all graphs represent SD

The above results indicate that DC-SIGNR enhances colon cancer cell adhesion, migration and invasion. The liver is the main site of DC-SIGNR production, and metastasis to the liver is a characteristic step in the development of colon cancer. Thus, we wanted to explore the role of mouse DC-SIGNR in this process. It has been demonstrated that plasmid DNA can be efficiently delivered to the liver by a hydrodynamic tail vein procedure, which has allowed for the elucidation of the roles of genes in disease development in animal models [28]. We first injected two different mouse DC-SIGNR shRNA plasmids and a control shRNA plasmid into the tail vein of nude mice. The effect of the two mouse DC-SIGNR shRNAs was determined by RT-PCR and Western Blot analysis after 3 days (Fig. 3a, b). Compared with the control shRNA, both of the DC-SIGNR shRNAs could reduce the expression of mouse DC-SIGNR. In addition, mouse DC-SIGNR shRNA a was more efficient than mouse DC-SIGNR shRNA b. Thus, we chose to use mouse DC-SIGNR shRNA a for our subsequent studies. We also studied the expression of mouse DC-SIGNR in different organs after injection with the mouse DC-SIGNR shRNA plasmid (Additional file 5: Figure S2A and B). The results showed that the mouse DC-SIGNR shRNA plasmid significantly decreased the expression of mouse DC-SIGNR in the liver, whereas the gene expression in other organs, such as spleen and lung, did not change after injection with the mouse DC-SIGNR shRNA plasmid. We determined that the decreased expression of mouse DC-SIGNR could be detected 3 days after the mouse DC-SIGNR shRNA plasmid tail vein injection. The level of mouse DC-SIGNR gene expression was dependent on the amount of mouse DC-SIGNR shRNA plasmid that was injected. Thus, we used different doses (10, 30, 50, and 60 μg) of the mouse DC-SIGNR shRNA plasmid for the tail vein injection experiment (Fig. 3c). According to the results, injecting 60 μg of the mouse DC-SIGNR shRNA plasmid per mouse was more efficient than the other doses. Consequently, we chose 60 μg as the optimal dose of the mouse DC-SIGNR shRNA plasmid for further study of the suppression of mouse DC-SIGNR in the mouse liver. We evaluated the expression of mouse DC-SIGNR over time after the injection of 60 μg of the mouse DC-SIGNR shRNA (Fig. 3d). The shRNA had an effect by the third day after the tail vein injection, and on the fifth and seventh days there was a distinct decrease in mouse DC-SIGNR expression. However, on the tenth day there was no statistical effect on the expression of mouse DC-SIGNR. These results suggest that 60 μg of the mouse DC-SIGNR shRNA can significantly decrease the expression of mouse DC-SIGNR and that its effects may last for 10 days in vivo.

Next, we examined whether mDC-SIGNR could bind to colon cancer cells. LoVo and LS174T colon cancer cells were incubated with frozen liver sections that were obtained from mice 3 days after treatment with either 60 μg of the mouse DC-SIGNR shRNA plasmid or with the control shRNA plasmid by tail vein injection (Fig. 3e, f). The results showed that compared with the control shRNA group, the adhesion of the colon cancer cells to the mouse liver tissues that had been treated with mouse DC-SIGNR shRNA was significantly decreased. These data demonstrate that the knockdown of mouse DC-SIGNR affects the adhesion of human colon cancer cells to liver tissues.

We then treated mice with 60 μg of the mouse DC-SIGNR shRNA plasmid or a control shRNA plasmid via tail vein injection. After 3 days, 5 × 10^6^ LoVo or LS174T cells were injected into the spleens of nude mice. The mouse DC-SIGNR shRNA or control shRNA were re-administered by tail vein injection every 10 days. The results showed that mouse DC-SIGNR shRNA significantly attenuated the metastasis of both LoVo and LS174T cells to the liver in comparison to the control shRNA (Fig. 3g). In addition, the mouse DC-SIGNR shRNA had no influence on the local growth of the colon cancer cells. Kaplan-Meier survival curves were generated to compare the condition of mice injected with the two different cell lines and treated with either the mouse DC-SIGNR or the control shRNA. We found that for mice injected with LoVo cells, the survival percentage of mice in the mouse DC-SIGNR shRNA group was significantly higher than in the control shRNA group (Fig. 3h). The same results were obtained for mice injected with LS174T cells. Moreover, we observed the histological changes in the liver surrounding metastatic foci by H&E staining, which revealed that knocking down mouse DC-SIGNR significantly decreased the potency of LoVo cell liver metastasis. However, there was no difference in spleen tumors between the mouse DC-SIGNR shRNA group and the control group (Fig. 3i). Furthermore, we measured the mean tumour volumes in each group and calculated the numbers of metastatic foci in the liver and spleen (Fig. 3j, k). The tumor volumes in the mouse DC-SIGNR shRNA group were smaller, and the mice developed fewer micrometastatic foci in liver tissue in the mouse DC-SIGNR shRNA experimental group compared with the control group. However, the mouse DC-SIGNR shRNA plasmid did not exert effects on the local growth of colon cancer cells. These data imply that mouse DC-SIGNR knockdown inhibits colon cancer liver metastasis in vivo.

### Human DC-SIGNR increases liver metastasis in vivo

**Fig. 4:**
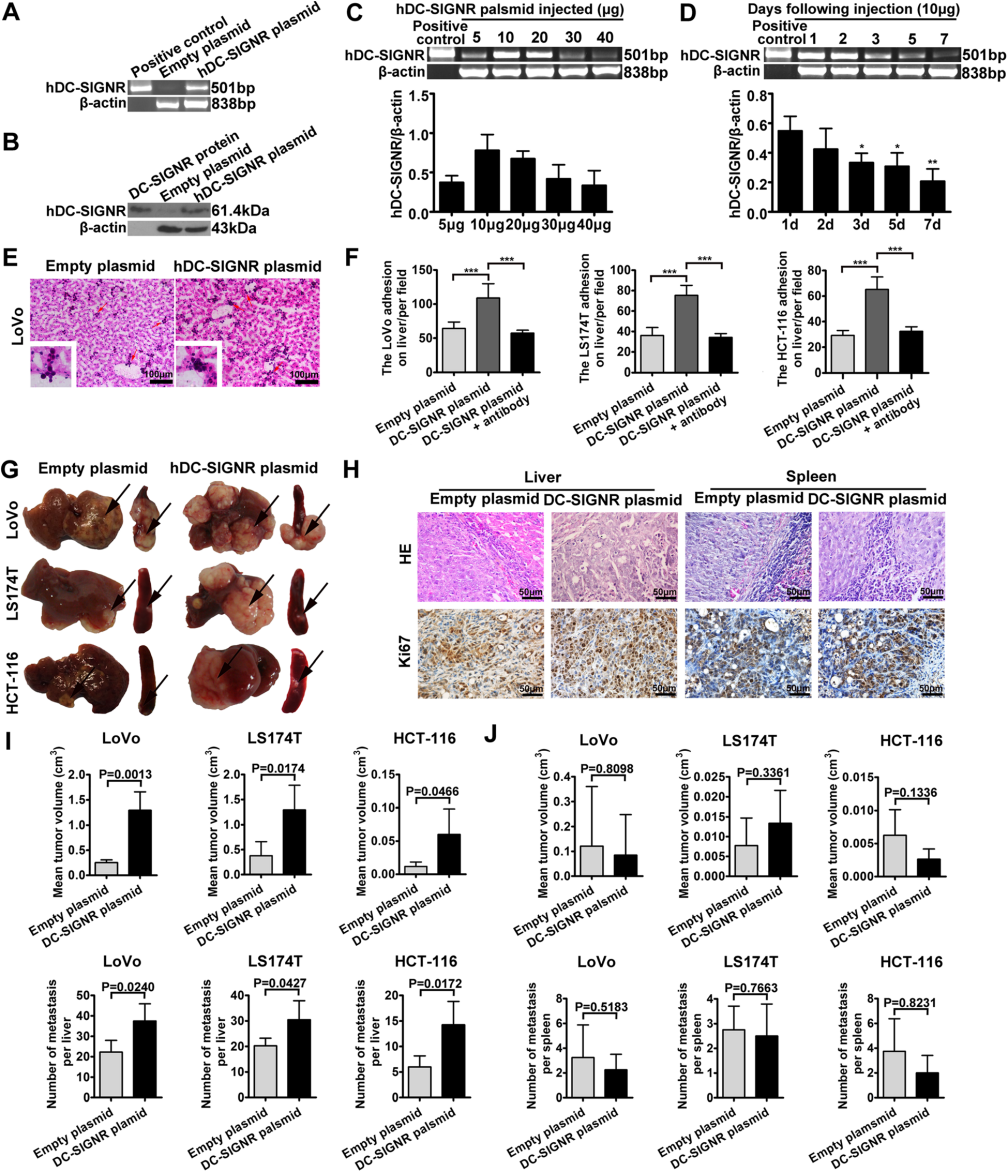
Human DC-SIGNR is involved in colon cancer liver metastasis in vivo. **a** The impact of delivering a human DC-SIGNR expression plasmid to mouse livers via tail vein injection was determined by RT-PCR analysis. Naked human DC-SIGNR plasmid DNA was used as a positive control. β-actin served as a loading control. **b** The influence of the human DC-SIGNR expression plasmid was detected by a Western Blot assay. The recombinant DC-SIGNR protein acted as a positive control. **c** The human DC-SIGNR expression levels were observed after mice were treated with different doses of the DC-SIGNR plasmid. The human DC-SIGNR levels were determined by RT-PCR (*upper panel*), and the relative expression of DC-SIGNR was quantified from three triplicate experiments (*lower panel*). The greyscale analysis of the DC-SIGNR bands was normalized to β-actin using Gelpro 32. **d** RT-PCR analysis of the human DC-SIGNR expression level in mice treated with 10 μg of the plasmid for the indicated lengths of time. *P < 0.05, **P < 0.01 vs the 1 d group. **e** Liver tissues were taken from mice one day after injection with the DC-SIGNR plasmid or an empty plasmid via tail vein. The adhesion of LoVo cells to frozen liver sections was detected by H&E staining. The red arrow marks the adherent colon cancer cells. ***P < 0.001. **f** DC-SIGNR expression can significantly promote the adhesion of the three types of colon cancer cells to liver tissues from mice treated with the DC-SIGNR expression plasmid by tail vein injection compared with the control group, and the interaction can be inhibited by a DC-SIGNR antibody. The cells that had adhered to the liver tissues were counted. **g** Images showing representative tumors in liver and spleen (*n* = 4 in each group). Human DC-SIGNR could increase the potency of liver metastasis in mice compared with an empty plasmid but had no influence on the local tumor size. **h** Representative images of H&E and Ki67 staining of tumour tissues in liver and spleen are shown. **i** The graph shows the mean volume of tumors and the number of metastases in the liver. **j** The volume of tumors and the number of metastatic spleen foci was measured in each group. The *error bars* in all graphs represent SD

The above findings demonstrate that mouse DC-SIGNR is involved in colon cancer liver metastasis. However, the role of human DC-SIGNR in this process has not been studied. Thus, we also used hydrodynamic injection to induce effective expression of exogenous human DC-SIGNR in mouse livers. The effect of the human DC-SIGNR expression plasmid was examined by RT-PCR and Western Blot analysis after 1 day (Fig. 4a and b). We found that human DC-SIGNR was expressed in the mouse liver after a tail vein injection with the human DC-SIGNR plasmid. We then injected different doses of the human DC-SIGNR plasmid, and consequently identified 10 μg as the optimal dose for human DC-SIGNR expression (Fig. 4c). We injected mice with 10 μg of the human DC-SIGNR plasmid and observed the gene expression in the liver over time. We found that the human DC-SIGNR plasmid had obvious effects on the first and second days after injection. However, the gene expression was gradually attenuated, and on the seventh day, we observed only faint DC-SIGNR expression in the liver tissues (Fig. 4d). These findings indicate that human DC-SIGNR can be expressed in mouse livers by human DC-SIGNR plasmid tail vein injection and that its effect may last for 7 days.

In addition, we detected whether human DC-SIGNR could adhere to liver tissue in mice. We incubated LoVo, LS174T, and HCT-116 colon cancer cells with frozen liver tissues from mice treated with the human DC-SIGNR plasmid or an empty plasmid for 1 day. We found that there were significantly more cells adhered to the liver tissues from the DC-SIGNR plasmid group compared with the control group, and the adhesive actions were inhibited by an anti-DC-SIGNR antibody (1:500, Abcam, USA) (Fig. 4e, f). These studies show that the expression of human DC-SIGNR in mouse livers induced by hydromechanical plasmid application could feasibly attract colon cancer cells to adhere to the liver tissue.

Thus, we have demonstrated that human DC-SIGR could bind to liver tissue in mice, and it is necessary to explore whether human DC-SIGNR is also related to colon cancer liver metastasis. Nude mice were first administered 10 μg of the human DC-SIGNR plasmid or an empty plasmid by tail vein injection. After 1 day, 5 × 10^6^ LoVo, LS174T, or HCT-116 cells were injected into the spleens of the mice. The injections of the human DC-SIGNR plasmid or the empty plasmid were repeated every 7 days. Compared with the control groups, the liver metastases volume was much larger in the mice injected with the three colon cancer cell lines and treated with the human DC-SIGNR plasmid (Fig. 4g). In addition, there was no difference in the volume of the spleen tumours between the human DC-SIGNR plasmid group and the control group. Next, we observed the histological changes in the liver surrounding metastatic foci and local spleen tumours by H&E staining and immunohistochemical staining of Ki-67, CEA, CK20, and CK7 (Fig. 4h; Additional file 5: Figure S2C). The H&E staining of the metastases confirmed their homology with the colon cancer cells, and there were more colon cancer cells in the human DC-SIGNR plasmid group in comparison to the empty plasmid group. Ki67 staining indicated that more hyperproliferative Ki67^+^ tumour cells were found in the liver tissue in the human DC-SIGNR plasmid group compared with the control group. Furthermore, we also measured the tumor volume and counted the numbers of metastatic foci in the liver and spleen for the groups injected with the different colon cancer cell lines (Fig. 4i, j). Compared with the empty plasmid group, the liver tumors grew much larger in the human DC-SIGNR plasmid group. In addition, the human DC-SIGNR plasmid surprisingly induced more micrometastatic liver foci compared to the empty plasmid. Additionally, in the mice injected with the three cell lines, there were no differences in the tumor volumes and the number of tumors per spleen between the group treated with the human DC-SIGNR plasmid and the group treated with the empty plasmid. In sum, these data confirm that DC-SIGNR may participate in colon cancer cell metastatic progression in the liver.

### DC-SIGNR significantly induces the expression of metallothioneins in colon cancer cells

**Fig. 5:**
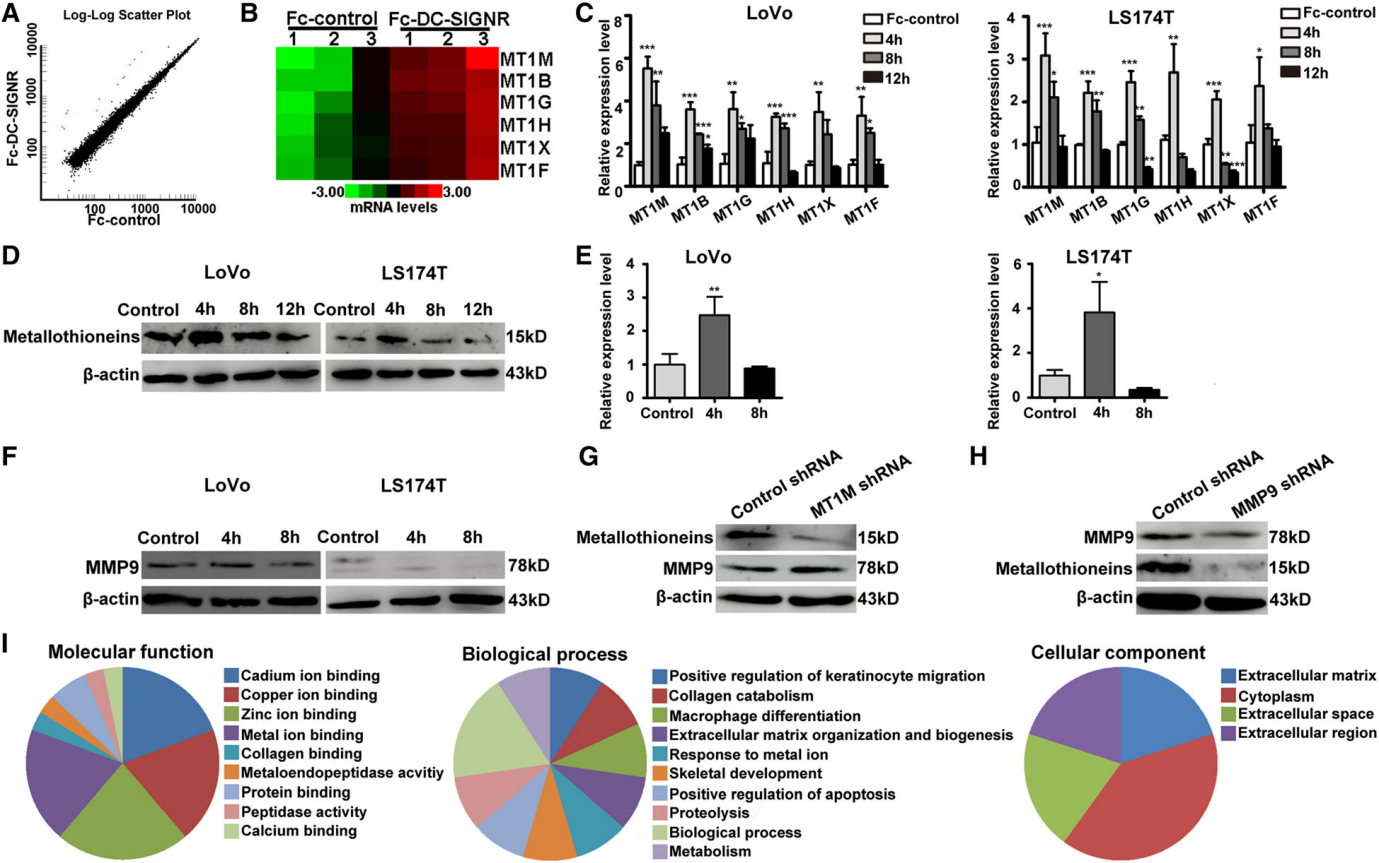
DC-SIGNR controls the expression of metallothioneins and MMP9. **a** Scatter plot of LoVo cells treated with the DC-SIGNR protein or control IgG for 4 h. The plot shows the different gene signal values between the two groups. The genes marked in *red* were upregulated, and the genes labelled in *black* were unchanged. **b** Hierarchical clustering of LoVo cells incubated with the DC-SIGNR protein or control IgG for 4 h, according to six differentially expressed genes (*Q* value ≤0.05 and fold change ≥2). The *coloured lines* of the dendrogram represent the support for each clustering. *Green* represents a low relative level and *red* indicates a high relative level of gene expression. **c** Differential expression of metallothioneins in two colon cancer cell lines (LoVo and LS174T) analysed using real-time PCR. The cells were treated with 5 μg/ml DC-SIGNR or control IgG in complete media for 4, 8, and 12 h. The mRNA expression of the genes was normalized to the GAPDH level. **d** The expression of metallothioneins in LoVo and LS174T cells treated with DC-SIGNR protein or control IgG (4, 8, and 12 h) was detected by Western Blot. β-actin was used as a control. **e** Real-time PCR was used to determine MMP9 expression in LoVo cells and LS174T cells that were incubated with the DC-SIGNR protein or control IgG for 4 h and 8 h. **f** A Western Blot assay was used to detect the expression of MMP9 in the two colon cancer cell lines treated with the DC-SIGNR protein or control IgG for 4 and 8 h. **g** The expression of MMP9 and metallothioneins was analysed by Western Blot in LoVo cells after infection with lentiviral MT1M shRNA and control shRNA. **h** The levels of metallothioneins and MMP9 were detected in LoVo cells after MMP9 knockdown by Western Blot. **i** Gene Ontology analysis of metallothioneins and MMP9. The analysis included three parts: molecular function, biological process and cellular component. The *error bars* in all graphs represent SD, and each experiment was repeated three times

To reveal the molecular alterations induced by DC-SIGNR in colon cancer cells, we utilized the profiling data from the Affymetrix Genechip PrimeView Human Gene Expression Array. LoVo cells were incubated with the DC-SIGNR protein or control IgG for 4 h. A total of 17,938 genes passed the filtering criteria and were further analysed. Among these genes, only the expression profiles of metallothionein family members (MT1M, MT1B, MT1G, MT1H, MT1X, MT1F) could be readily distinguished between DC-SIGNR protein-treated cells and the control cells (Fig. 5a). Interestingly, there was no difference in the genes related tumour metastasis, such as MET, DRG1, or SMAD7 (Table 1). Notably, the average and median expression values of metallothioneins were higher in the Fc-DC-SIGNR test group than in the Fc-control group (Fig. 5b; Additional file 6: Table S1). To validate the array results, semi-quantitative RT-PCR and Western Blot analysis were performed (Fig. 5c, d; Additional file 7: Figure S3A and B). We detected the expression of metallothioneins in LoVo and LS174T cells incubated for different lengths of time with the DC-SIGNR protein or control IgG. Consistent with the microarray data, both cell lines treated with the DC-SIGNR protein showed a more intense signal than the control cells after 4 h of incubation. In addition, the expression of metallothioneins gradually decreased in a time-dependent manner in the Fc-DC-SIGNR group.

**Table 1:** Up-regulated and part of non-dysregulated genes by DC-SIGNR stimulated of LoVo cells

Affymetrix gene ID	Fold change	*Q* value	Symbol	Gene description
11728398_x_at	13.9704	0.0000	MT1M	Metallothionein 1M
11730028_at	8.5857	0.0000	MT1B	Metallothionein 1B
11717385_a_at	7.1304	0.0000	MT1G	Metallothionein 1G
11731857_x_at	5.7220	0.0000	MT1H	Metallothionein 1H
11757581_x_at	5.0677	0.0000	MT1X	Metallothionein 1X
11721877_s_at	4.8745	0.0000	MT1F	Metallothionein 1F
11723326_at	1.8636	0.0000	CA9	Carbonic anhydrase IX
11716408_a_at	1.025	39.5270	MET	Met proto-oncogene
11763609_a_at	0.8917	124.9533	DRG1	Developmentally regulated GTP binding protein 1
11725514_a_at	0.9209	124.9533	SMAD7	SMAD family member 7
11734015_a_at	1.3274	39.5271	SMAD2	SMAD family member 2
11725363_s_at	1.1185	39.5271	SMAD4	SMAD family member 4
11721124_s_at	0.9999	124.9533	MMP11	Matrix metallopeptidase 11(stromelysin 3)
11725989_x_at	1.0338	39.5271	MMP14	Matrix metallopeptidase 14(membrane-inserted)
11727281_a_at	1.0732	39.5271	MMP28	Matrix metallopeptidase 28
11748126_a_at	1.2275	39.5271	TIMP2	TIMP metallopeptidase inhibitor 2
11724904_at	0.8562	124.9533	TIMP4	TIMP metallopeptidase inhibitor 4
11750190_x_at	1.0254	39.52701	CD44	CD44 molecule (Indian blood group)
11727668_a_at	0.9311	124.9533	MTA1	Metastasis associated 1
11758421_s_at	0.988	124.9533	PTEN	Phosphatase and tensin homolog
11721226_x_at	1.1754	39.5271	MTSS1L	Metastasis suppressor 1-like
11745021_a_at	0.8883	124.9533	MYC	v-myc myelocytomatosis viral oncogene homolog (avian)
11728523_a_at	1.2524	39.5271	MYCL1	v-myc myelocytomatosis viral oncogene homolog 1, lung carcinoma derived (avian)
11727802_s_at	1.2701	39.5271	MYCBP	c-myc binding protein
11717155_a_at	1.0641	39.5271	NME1	Non-metastatic cells 1, protein (NM23A) expressed in NME1-NME2 readthrough
11718347_a_at	0.9329	124.9533	S100P	S100 calcium binding protein P
11725371_s_at	0.9601	124.9533	VEGFA	Vascular endothelial growth factor A
11758873_a_at	1.4568	39.5271	HPSE	Heparanase
11754925_a_at	1.1226	39.5271	SYK	Spleen tyrosine kinase

Several studies have reported that metallothioneins are capable of mediating MMP9 expression [29–31]. Our results have demonstrated that the DC-SIGNR protein could significantly increase the expression of metallothioneins after 4 h of incubation. Thus, we wanted to know whether DC-SIGNR could alter MMP9 expression. Interestingly, we found that DC-SIGNR also increased MMP9 expression in LoVo and LS174T cells after 4 h of incubation, and this gene was not analysed by the gene expression array (Fig. 5e, f). Furthermore, we explored the relationship between MMP9 and metallothioneins in colon cancer cells. As a result, we found there was no obvious change in MMP9 protein expression after MT1M (which was upregulated most obviously of the metallothioneins) knockdown in LoVo cells (Fig. 5g). Silencing MMP9 significantly decreased the expression of metallothioneins in LoVo cells as determined by Western Blot analysis (Fig. 5h). In addition, we also reviewed syndicated bioinformatics using the Molecule Annotation System 3.0 and found that metallothioneins and MMP9 have many binding sites, are involved in many biological processes, and are expressed mainly in the cytoplasm (Fig. 5j). This evidence reveals that DC-SIGNR regulates the expression of metallothioneins by upregulating MMP9 during colon cancer liver metastasis.

### Increased levels of soluble DC-SIGNR were detected in colon cancer patients with liver metastasis

**Fig. 6:**
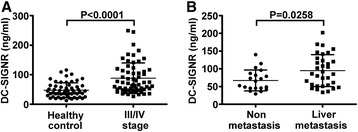
Soluble DC-SIGNR is significantly upregulated in colon cancer patients with liver metastasis. The serum levels of DC-SIGNR were detected by an enzyme-linked immunosorbent assay. **a** The scatter plot shows the comparison of serum DC-SIGNR levels between patients with stage III/IV colon cancer and healthy individuals. The level of serum DC-SIGNR was significantly higher in patients with stage III/IV colon cancer compared with healthy controls (*P* < 0.0001). **b** Comparison of DC-SIGNR levels in colon cancer patients with liver metastasis and those without metastasis. The serum DC-SIGNR level was statistically higher in colon cancer patients with liver metastasis than those without metastasis (*P* = 0.0258). The *error bars* in all graphs represent SD

Cell adhesion molecules are important mediators in the process of tumor growth and metastasis. They are released into circulation or internalized rapidly under shear conditions. In addition, increased serum levels of adhesion molecules were reported to be correlated with tumour metastasis [32]. In this study, we examined the serum levels of DC-SIGNR in colon cancer patients to detect the relationship between soluble DC-SIGNR and colon cancer liver metastasis. We investigated 138 participants in total, and the serum levels of DC-SIGNR in colon cancer patients (*n* = 81) and healthy controls (*n* = 57) were analysed by a standard sandwich ELISA (Table 2). The results showed that serum DC-SIGNR levels were significantly different between colon cancer patients in stage III/IV (88.16 ± 51.78 ng/ml) and healthy individuals (47.72 ± 24.94 ng/ml) (*P* < 0.0001) (Fig. 6a). In addition, we detected that the serum concentrations of DC-SIGNR were significantly higher in colon cancer patients with liver metastasis (94.81 ± 44.99 ng/ml) than in patients without metastasis (67.20 ± 29.67 ng/ml) (*P* = 0.0258) (Fig. 6b). These results validate that soluble DC-SIGNR may be correlated with the process of colon cancer liver metastasis.

**Table 2:** Clinical data of the colon cancer patients in ELISA study

Clinical data	Cases	Proportion	sDC-SIGNR (ng/ml) median (range)
Gender			
Female	32	39.51%	72.88 (33.46–245.0)
Male	49	60.49%	89.73 (26.81–249.3)
Age (year)			
≧60	52	64.20%	62.25 (29.49–249.3)
<60	29	35.80%	74.76 (26.81–245.0)
Stage			
I/II	20	24.69%	58.90 (29.49–140.0)
III/IV	61	75.31%	70.97 (26.81–249.3)
Type of cancer			
Colon	42	51.85%	67.51 (33.83–249.3)
Rectal	39	48.15%	66.53 (26.81–202.2)
Metastatic site			
No	20	24.69%	58.90 (29.49–140.0)
Liver	33	40.74%	86.26 (33.83–202.2)
Other	28	34.57%	59.71 (26.81–249.3)

## Discussion

In this study, we have demonstrated that DC-SIGNR plays a functional role in promoting colon cancer metastasis. Our results showed that the DC-SIGNR protein dramatically induced colon cancer cell adhesion, migration and invasion properties in vitro. Mouse DC-SIGNR and human DC-SIGNR are both involved in colon cancer liver metastasis in vivo. Furthermore, we showed that DC-SIGNR promoted the liver metastasis of colon cancer cells through a novel regulatory pathway in which DC-SIGNR notably increased the expression of metallothionein isoforms and MMP9. Consistent with our observation, the serum levels of DC-SIGNR were significantly higher in colon cancer patients with liver metastasis than those without metastasis. In sum, we have clearly demonstrated that DC-SIGNR is a critical mediator of colon cancer liver metastasis.

**Fig. 7:**
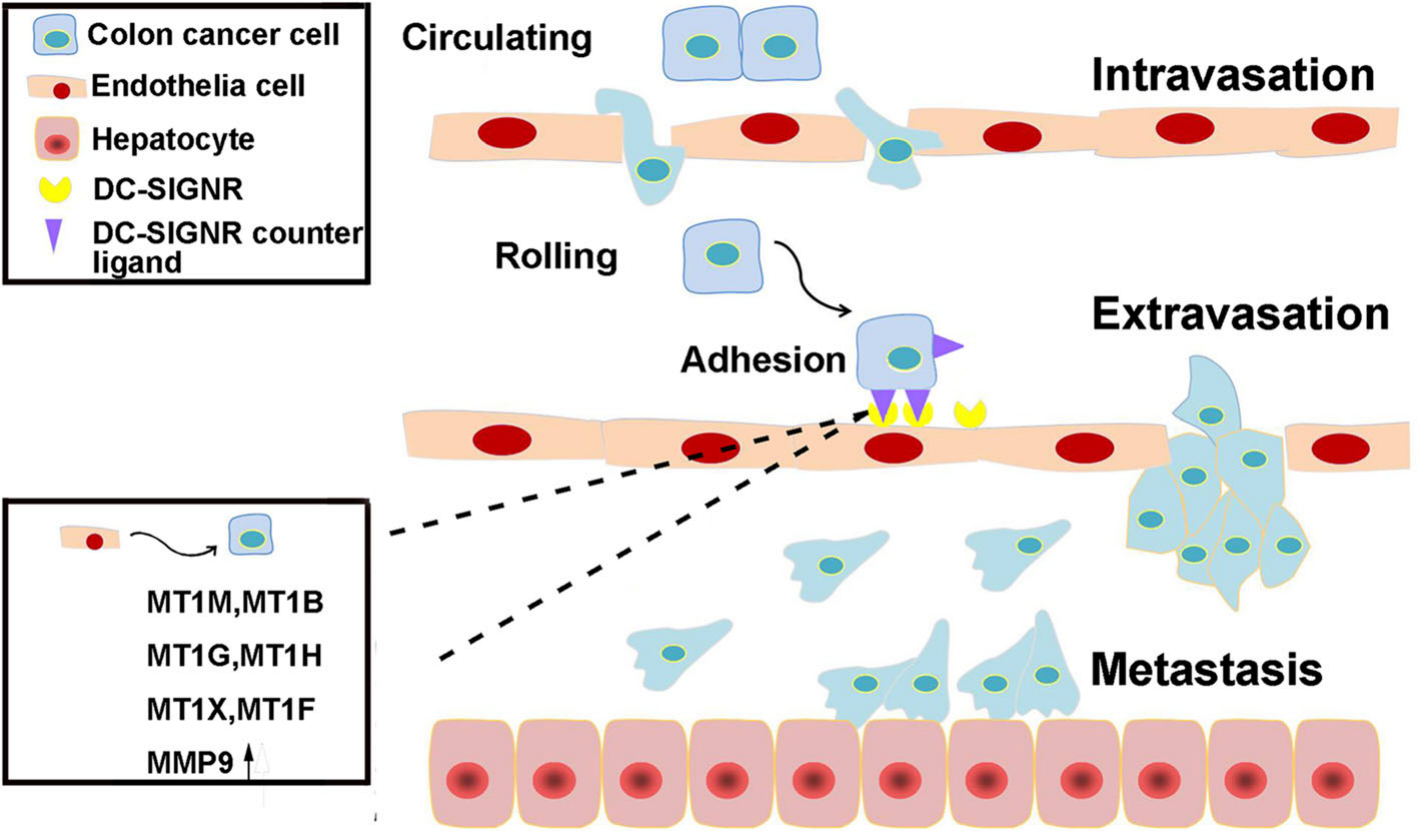
Liver metastasis formation. Colon cancer cells detach from the primary sites, circulate in the surrounding tissues, and enter the blood vessels (intravasation). The cells adhere to endothelial cells through the interaction between DC-SIGNR as a receptor on the surface of endothelial cells and the receptor ligand expressed on colon cancer cells. DC-SIGNR increases the expression of MT1M, MT1B, MT1G, MT1H, MT1X, MT1F, and MMP9 in colon cancer cells. Then, the cells are able to extravasate, migrate, invade into the nearby liver, and form the metastatic tumour

Here, we have found that serum DC-SIGNR levels in colon cancer patients with liver metastasis were higher than in those without metastasis. Importantly, it is necessary to know whether colon cancer cells express DC-SIGNR. We did not detect DC-SIGNR expression in the three colon cancer cell lines used in this study by either flow cytometry or Western Blot analysis (Additional file 8: Figure S4, A and B). In addition, our laboratory has reported that immunohistochemical staining for DC-SIGNR was negative in matched colon tissues and tumour stroma and was only faintly positive between the colon cancer foci [13]. Thus, we speculated that DC-SIGNR, which is expressed in the liver, triggers colon cancer cell adhesion, contributes to increased liver endothelial cell permeability, and enables the transendothelial migration and invasion of colon cancer cells (Fig. 7). The expression of DC-SIGNR was increased and DC-SIGNR was released into the bloodstream once colon cancer cells appeared in the liver. This may explain the upregulated serum levels of soluble DC-SIGNR in colon cancer patients with liver metastasis.

DC-SIGNR is a trans-membrane protein, which commonly consist of three parts: extra-cellular, trans-membrane, and intra-cellular domains. There is a neck domain and a carbohydrate-recognition domain (CRD) in the extra-cellular domain of DC-SIGNR. Reports have shown that mouse DC-SIGNR has significant homology to human DC-SIGNR in the CRD and transmembrane domain [33]. Studies of the ligand binding properties indicate that mouse DC-SIGNR interacts with a series of fucosylated oligosaccharides (Lewis^a^, Lexwis^b^, Lewis^x^, and Lewis^y^) and mannose structures as an adhesion receptor. In addition, mouse DC-SIGNR also binds to human ICAM-2, which promotes lymphocyte migration into the white pulp [34]. Moreover, mouse DC-SIGNR is also abundantly expressed in the liver and lymph nodes, similar to human DC-SIGNR. Thus, mouse DC-SIGNR is very similar to human DC-SIGNR. We have demonstrated that mouse DC-SIGNR was involved in colon cancer liver metastasis, and down-regulating the expression of mouse DC-SIGNR could reduce the metastasis of the tumour. However, considering both structure and expression site, there is no single orthologue of DC-SIGNR in mice that is strictly identical to human DC-SIGNR. In contrast to the high similarity in the CRD region, the mouse DC-SIGNR has a much shorter neck domain compared to human DC-SIGNR. There is a 191-amino acid neck domain encoded by a single exon in human DC-SIGNR. However, the mouse DC-SIGNR neck domain has only 116 amino acids and is encoded by four small exons [35]. This is probably a consequence of a lesser degree of homology between mouse DC-SIGNR and human DC-SIGNR. The expression of mouse DC-SIGNR is similar but not identical to that of human DC-SIGNR. Mouse DC-SIGNR is also expressed by macrophages in the spleen marginal zone and lymph node medulla and in the peritoneal cavity of BALB/C mice [36]. It seems that mouse DC-SIGNR has attributes of both human DC-SIGNR and DC-SIGN [37]. However, mouse DC-SIGNR cannot completely replace human DC-SIGNR. Thus, we also observed the role of human DC-SIGNR in colon cancer liver metastasis. Consequently, we found that human DC-SIGNR also participated in colon cancer liver metastasis and that increasing the expression of human DC-SIGNR promoted the occurrence of colon cancer liver metastasis.

Moreover, we also demonstrated that DC-SIGNR regulates the expression of metallothioneins and MMP9. Increased expression of metallothioneins is a prognostic indicator of poor survival for many cancers, such as gastric cancer, non-small lung cancer, and breast cancer [20, 38, 39]. In addition, metallothioneins are also reported to correlate with tumour metastasis. In squamous cell carcinoma of the oesophagus, metallothioneins could predict lymph node status and distant metastasis and were correlated with cell proliferation potential [40]. The overexpression of metallothioneins was statistically more common in primary cutaneous malignant melanoma with haematogenous metastases, independent of Breslow tumour thickness [41]. For colon cancer, the overexpression of metallothioneins was a prognostic indicator for the poor overall survival of patients with colorectal cancer, independent of some main clinicopathological parameters [42]. To the best of our knowledge, this study is the first to analyse the expression profiles of colon cancer cells treated with the DC-SIGNR protein or control IgG using a microarray. Interestingly, our data showed that metallothioneins (MT1M, MT1B, MT1G, MT1H, MT1X, and MT1F) significantly increased after 4 h of DC-SIGNR protein treatment and then decreased in a time-dependent manner. Time-course analyses of metallothionein expression have also been reported by other researchers [43–45]. For example, nickel significantly increased the transcript levels of MT2A by 2 h, with the maximal induction occurring at 4 h after exposure, followed by a subsequent decrease in expression [43]. Ren et al. demonstrated that cadmium-induced metallothionein mRNA expression was time-dependent in rats, showing that the mRNA expression levels were substantially increased after Cd treatment, peaked at 3 h, and then declined. In addition, the mRNA levels of metallothioneins in Sertoli cells peaked at 6 h [44]. WAKIDA et al. found that MT-1 mRNA expression was significantly increased 6 h after a middle cerebral artery occlusion and decreased at 12 and 24 h in the wild-type mouse brain [45].

In addition, several studies have reported the function of metallothioneins in the regulation of MMP9 [29–31]. MMP9, a gene in the family of zinc-dependent enzymes and capable of degrading various extracellular matrix components, is associated with colon cancer liver metastasis [46, 47]. Ondrej Zitka et al. [29] showed that the activity of MMP9 was higher in the presence of metallothioneins compared with an experimental collagen-MMP9 mixture by chip-based gel electrophoresis, which was similar to the temperature effect. Metallothionein-2A, a member of the metallothioneins, could promote breast cancer cell invasion by increasing the expression level of MMP9. MT-2A activated the binding sites in the MMP9 promoter region via modulating AP-1 and NF-κB [30]. Another member, MT1E, could modulate the motility and invasion of a human glioma cell line by upregulating MMP9 as well, and this interaction was also related to an association between MT1E and NF-κB [31]. In previous studies, possibly due to the high affinity of metallothioneins for heavy metal zinc ions, it was shown that metallothioneins can induce the activities of zinc-dependent regulatory proteins, such as enzymes and zinc-finger transcription factors. For MMP9, it may be postulated that zinc ions present in the metallothioneins activate MMP9, which then degrades various extracellular matrix proteins. Therefore, since we demonstrated that the expression of metallothioneins increased as a result of DC-SIGNR simulation, we also wondered whether DC-SIGNR treatment had an effect on MMP9 expression. Surprisingly, we found that the expression of MMP9 was increased after 4 h of incubation with the DC-SIGNR protein and then decreased in the next 4 h. Similar to metallothioneins, MMP9 degrades collagen with high efficiency and it is also expressed in a time-dependent manner [48–50]. Warren’s findings indicated that uterine MMP9 increased in response to oestrogen at 2–4 h and then decreased, reaching basal levels at 8 h [48]. Gilet et al. reported that aldosterone increased MMP9 mRNA levels in HL-60 cells after 1.5 h of incubation, and the MMP9 expression peaked at 6 h, and then degraded by 24 h [49]. Zhang et al. demonstrated that LPS triggers a rapid continuous increase in MMP9 expression in blood samples during 1–3 h of incubation, but there was no MMP9 detected after 3 h [50]. In our study, colon cancer cells flowed into blood circulation and arrested the site of liver by adhering to the sinusoidal cells. DC-SIGNR, which was expressed in liver endothelial cells, increased the expressions of metallothioneins and MMP9 in colon cancer cells at 4 h with high activity.

Actually, cellular adhesion molecules play important roles in tumor metastasis. The adhesive interaction of specific adhesion molecules and receptors are the intermediate procedures of the metastatic cascade. DC-SIGNR, as it has a high affinity for ICAM-3, can capture the mannose glycans present on HIV-1 gp120 and promote HIV-1 infection of T cells in trans [51]. DC-SIGNR expressed in sinus endothelial cells was demonstrated to form a continuous lining that mediated the binding to ICAM-3 on T lymphocytes during their migration out of the lymph nodes during an immune response [52]. Our results showed that DC-SIGNR interacted with colon cancer cells in a dose- and calcium-dependent manner. Colon cancer cells exhibited a significantly and specifically reduced adhesion to liver tissues that were extracted from mice injected with the mouse DC-SIGNR shRNA plasmid compared with tissues that were taken from mice injected with the control shRNA plasmid. The number of colon cancer cells adhering to liver tissues was augmented when the mice were injected with a human DC-SIGNR expression plasmid. And the interaction could be inhibited by the DC-SIGNR antibody which acted on the tandem-repeated neck domain of DC-SIGNR. For DC-SIGNR, the neck region is essential for lectin tetramerization. This neck structure may constitute a rigid neck support for the CRD and change the angles and distances between the carbohydrate-binding sites within the each constituent monomer. Thus, the neck domain may have a significant impact on ligand binding. Thus, these results provide the first experimental evidence to date linking colon cancer cell adhesion to hepatic endothelial DC-SIGNR with the progression of liver metastasis.

In addition, cell migration is also an essential factor in tumour metastasis. In our study, we found that DC-SIGNR increased the expression of metallothioneins and MMP9. Metallothioneins are a family of cysteine-rich, low molecular-weight proteins. In addition, their expression in the tumour plays an important role in cell migration and therefore influences the metastatic ability of tumour. For example, Wu et al. reported that MT1E, one of the metallothioneins, was considered to be important in bladder cancer cell migration and tumour stage [53]. It has been demonstrated that metallthionein-3 induces migration, proliferation, and invasion in triple-negative breast cancer cells via the induction of metalloproteinase expression [54]. Another key molecular mediator of DC-SIGNR in colon cancer cells is MMP9. MMP9 production and secretion by tumor cells are vital factors involved in the promotion of metastasis. MMP9 can promote tumour progression by degrading the extracellular matrix and facilitating cell migration, invasion and metastasis [55]. In addition, silencing MMP9 expression decreases cell migration and invasion and induces cell aggregation [56, 57]. Our results also showed that MMP9 knockdown in LoVo cells could significantly decrease the expression of metallothioneins; however, there was no distinct effect on MMP9 expression after MT1M silencing. Thus, we concluded that DC-SIGNR, as an important regulator, promoted colon cancer cell migration, invasion and metastasis through the activation of metallothioneins by upregulating MMP9 expression.

## Conclusions

In summary, our study on DC-SIGNR indicates its novel role in colon cancer liver metastasis. DC-SIGNR may be a promising biomarker that provides a new therapeutic strategy for the treatment of colon cancer.

## Additional files

Additional file 1: Table S2. The table shows the clinical data of the colon cancer patients in DC-SIGNR ELISA study. (DOCX 24 kb)
Additional file 2: Figure S5. The standard curve of soluble DC-SIGNR. Human recombinant DC-SIGNR protein was for the standard sample and linear regression was completed successfully, *R* ^2^ = 0.9984. The OD value and corresponding concentration of sDC-SIGNR were listed. (TIF 394 kb)
Additional file 3: Table S3. The table exhibits the primer sequences used for quantitative real-time PCR. (DOCX 13 kb)
Additional file 4: Figure S1. DC-SIGNR did not promote colony formation in colon cancer cells. (A) The photographs indicated no influence of DC-SIGNR protein on cell proliferation efficiency of LoVo cells. (B) The histograms showed the clone cells number in respective cells. (TIF 1926 kb)
Additional file 5: Figure S2. Mouse DC-SIGNR expression in different organs and the tumor cells matched with colon cancer cells. (A-B) Mouse DC-SIGNR expression in different organs via mouse DC-SIGNR shRNA and control shRNA plasmid injection were detected by RT-PCR and Western Blot. These results showed that the method of hydrodynamic injection of mouse DC-SIGNR shRNA plasmid can significantly suppress mouse DC-SIGNR expression in the liver. However, there was no obvious effect on the mouse DC-SIGNR expression in spleen and lung. (C) Mice were injected with LoVo cells into their spleens after human DC-SIGNR plasmid tail vein injection. The liver and spleen tumor tissues were stained with colon cancer markers CEA, CK20, and CK7. The tumors were positive for CEA and CK20, and negative for CK7, which is a pattern exclusively in colon cancer. ****P* < 0.001. (TIF 2552 kb)
Additional file 6:Table S1. The table lists the gene expression profiling data analyzed by human gene expression array. (XLS 4041 kb)
Additional file 7: Figure S3. DC-SIGNR could not regulate the expressions of MET, DRG1 and SMAD7. (A-B) Colon cancer cells LoVo and LS174T were treated with DC-SIGNR protein or control IgG for 4 h. No differences in the expressions of MET, DRG1 and SMAD7 were detected by quantitative real-time PCR. (TIF 217 kb)
Additional file 8: Figure S4. DC-SIGNR could not be expressed in colon cancer cell lines. (A) The expression of DC-SIGNR was detected by flow cytometry. Results are presented as the percentage of three colon cancer cells expressing DC-SIGNR. (B) Western Blot was used to test the expression of DC-SIGNR in colon cancer cells. Recombinant DC-SIGNR protein was used as positive control. (TIF 463 kb)

## Abbreviation

GAPDH: Glyceraldehyde-3-phosphate dehydrogenase
hDC-SIGNR: Human DC-SIGNR
mDC-SIGNR: Mouse DC-SIGNR
PBS: Phosphate-buffered saline
