# Supplementary material for: Novel roles of DC-SIGNR in colon cancer cell adhesion, migration, invasion, and liver metastasis
Source: J Hematol Oncol. 2017 Jan 21;10:28. doi: 10.1186/s13045-016-0383-x (PMC5251210; doi:10.1186/s13045-016-0383-x)
Supplement: Additional file 1: Table S2. — The table shows the clinical data of the colon cancer patients in DC-SIGNR ELISA study. (DOCX 24 kb) [file 13045_2016_383_MOESM1_ESM.docx]

**Supplementary Table 2** Clinical data of the colon cancer patients in DC-SIGNR ELISA study

| No. | Gender/age | Tumor stage | Tumor  differentiation | sDC-SIGNR  (ng/ml) | CEA  (μg/l) | CA199  (U/ml) | AFP  (μg/l) |
| --- | --- | --- | --- | --- | --- | --- | --- |
| 1 | F/59 | II | Moderate | 65.22 | 3.71 | 22.31 | 1.7 |
| 2 | M/73 | II | Moderate | 48.83 | 2.59 | 28.39 |  |
| 3 | M/50 | II | - | 81.75 | 1.62 | 13.45 |  |
| 4 | F/48 | II | Well to moderate | 87.11 |  |  |  |
| 5 | F/55 | II | Moderate | 140.01 |  |  |  |
| 6 | M/59 | II | - | 36.66 | 2.78 | 0.01 |  |
| 7 | M/61 | II | Moderate | 103.34 | 2.7 | 11.52 |  |
| 8 | M/64 | II | - | 114.77 |  |  |  |
| 9 | M/53 | II | Moderate | 48.63 | 7.86 | 213.4 |  |
| 10 | F/60 | I | Well | 78.88 | 11.24 | 10.51 |  |
| 11 | M/74 | II | - | 73.17 | 7.89 | 1.34 | 3.3 |
| 12 | F/60 | II | Well | 96.15 | 11.24 | 10.51 |  |
| 13 | F/60 | II | Moderate | 45.72 |  |  |  |
| 14 | F/72 | II | Moderate | 72.00 | 5.19 | 8.98 |  |
| 15 | M/48 | II | Well-moderate | 45.29 |  |  |  |
| 16 | F/53 | II | Moderate | 45.29 |  |  |  |
| 17 | F/57 | II | Moderate | 52.58 |  |  |  |
| 18 | M/61 | II | Well | 29.49 |  |  |  |
| 19 | M/62 | II | Moderate | 37.16 |  |  |  |
| 20 | F/51 | II | Moderate | 42.03 |  |  |  |
| 21 | M/68 | III | Moderate | 63.19 | 3.64 | 14.62 | 2.17 |
| 22 | M/71 | III | Moderate | 53.59 | 0.97 | <3.5 | <1.0 |
| 23 | F/60 | III | Moderate | 45.67 | 38.67 | 33.39 | 2.6 |
| 24 | M/62 | III | Moderate | 70.97 |  |  |  |
| 25 | M/66 | III | Moderate | 155.13 | 1.34 | 14.29 |  |
| 26 | M/69 | III | Moderate to poor | 53.44 | 0.91 | 14.55 |  |
| 27 | M/42 | III | Moderate | 66.53 | 3.15 | 20.84 |  |
| 28 | F/52 | III | - | 182.04 | <0.5 | 10.32 | 3.8 |
| 29 | F/55 | III | Moderate to poor | 104.28 | 3.64 | 45.76 |  |
| 30 | F/64 | III | Moderate | 33.46 | 2.44 | 12.69 | 2.1 |
| 31 | M/53 | III | - | 26.81 | 19.93 | 69.28 |  |
| 32 | F/64 | III | Moderate | 77.69 | 0.91 | 7.44 | 0.7 |
| 33 | M/77 | III | Moderate | 44.03 |  |  |  |
| 34 | F/67 | III | Moderate | 110.89 | 46.62 | 37.45 | 5.3 |
| 35 | M/76 | III | Moderate | 43.09 | 2.7 | 17.47 |  |
| 36 | M/72 | III | Moderate to poor | 47.67 |  |  |  |
| 37 | F/46 | IV | - | 244.96 | 0.84 | 18.33 | 2.01 |
| 38 | M/80 | III | Moderate | 249.29 | 2.6 | 10.65 | 1.75 |
| 39 | F/61 | III | Moderate | 48.68 | 7.46 | 57.23 | 8.9 |
| 40 | M/64 | III | Moderate | 34.79 |  |  |  |
| 41 | M/62 | III | Moderate | 54.77 |  |  |  |
| 42 | F/63 | III | Moderate | 53.49 |  |  |  |
| 43 | M/69 | III | - | 74.02 |  |  |  |
| 44 | M/45 | III | Moderate | 59.94 | 0.79 | 10.88 | 4.1 |
| 45 | F/56 | III | Moderate | 63.01 | 0.97 | 33.13 |  |
| 46 | F/65 | III | Moderate | 35.84 | 0.96 | 8.11 |  |
| 47 | M/57 | IV | Poor | 92.18 | 3.81 | 3239.92 |  |
| 48 | M/65 | IV | Moderate | 59.49 | 138.18 | 2.46 |  |
| 49 | M/70 | IV | - | 113.94 | 11.67 |  |  |
| 50 | M/62 | IV | Moderate | 36.16 | 44.75 |  |  |
| 51 | M/81 | IV | - | 108.11 | 17.19 | 16.24 |  |
| 52 | F/76 | IV | Moderate | 33.83 | 72.09 | 79.08 | 4.52 |
| 53 | M/65 | IV | Moderate | 141.93 | 771.94 | 98.83 |  |
| 54 | M/62 | IV | Moderate | 146.78 | >60 | 354.12 | 4.84 |
| 55 | M/56 | IV | Well to moderate | 54.21 | 9.95 | 36.66 | 31.5 |
| 56 | M/63 | IV | - | 41.80 | 8.36 | 35.9 |  |
| 57 | M/60 | III | Moderate | 202.17 |  |  |  |
| 58 | F/65 | III | Poor | 86.26 | 895.93 | 2126.94 |  |
| 59 | F/66 | III | Moderate | 65.81 |  |  |  |
| 60 | M/74 | III | Well to moderate | 51.38 | 4.72 | 29.7 |  |
| 61 | M/68 | IV | Moderate | 47.86 |  |  | 0.94 |
| 62 | F/55 | IV | Moderate | 116.98 |  |  |  |
| 63 | M/49 | IV | Moderate | 169.36 | 58.06 | 35.78 | 4.36 |
| 64 | M/49 | IV | Moderate | 102.14 | 15.51 | 115.3 | 4.68 |
| 65 | F/53 | IV | - | 44.12 | 336.5 | 414.6 | 28.29 |
| 66 | F/53 | IV | Moderate | 85.34 | 2.91 |  |  |
| 67 | M/66 | IV | Moderate | 120.31 | 8.75 | 11.98 |  |
| 68 | F/78 | IV | - | 166.64 | 166.7 | 7.24 |  |
| 69 | M/69 | IV | Moderate | 92.09 | 43.95 | 242.7 |  |
| 70 | M/64 | IV | - | 61.32 | 87.68 | 65.67 | 9.01 |
| 71 | M/54 | IV | Moderate | 74.76 | 7.47 | 1.64 | 3.36 |
| 72 | M/61 | IV | Moderate to poor | 55.96 |  |  |  |
| 73 | F/79 | IV | - | 44.87 | 18.41 |  |  |
| 74 | M/72 | IV | Moderate to well | 145.71 | 67.84 | 22.24 | 1.26 |
| 75 | F/48 | IV | - | 129.84 | 1.25 | 9.01 | 2.99 |
| 76 | M/63 | IV | Moderate | 155.97 | 15.35 | 12.76 | 18.53 |
| 77 | M/62 | IV | Moderate | 80.01 | 75.69 | 72.43 | 10.88 |
| 78 | M/45 | IV | Moderate | 105.22 | 5.62 | 65.05 | 4.34 |
| 79 | M/71 | IV | Moderate | 50.88 | 16.38 | 12.59 | 3.43 |
| 80 | F/52 | IV | Moderate | 73.76 | >1000 | 28.92 | 12.73 |
| 81 | F/59 | IV | Moderate | 123.18 |  | 545.8 | 43.33 |

Note: F: female; M: male; CEA, carcinoembryonic antigen; CA199, carbohydrate antigen 199; AFP, Alpha Fetoprotein; -: not available.
