# Supplementary figures and images for: Novel roles of DC-SIGNR in colon cancer cell adhesion, migration, invasion, and liver metastasis
Source: J Hematol Oncol. 2017 Jan 21;10:28. doi: 10.1186/s13045-016-0383-x (PMC5251210; doi:10.1186/s13045-016-0383-x)

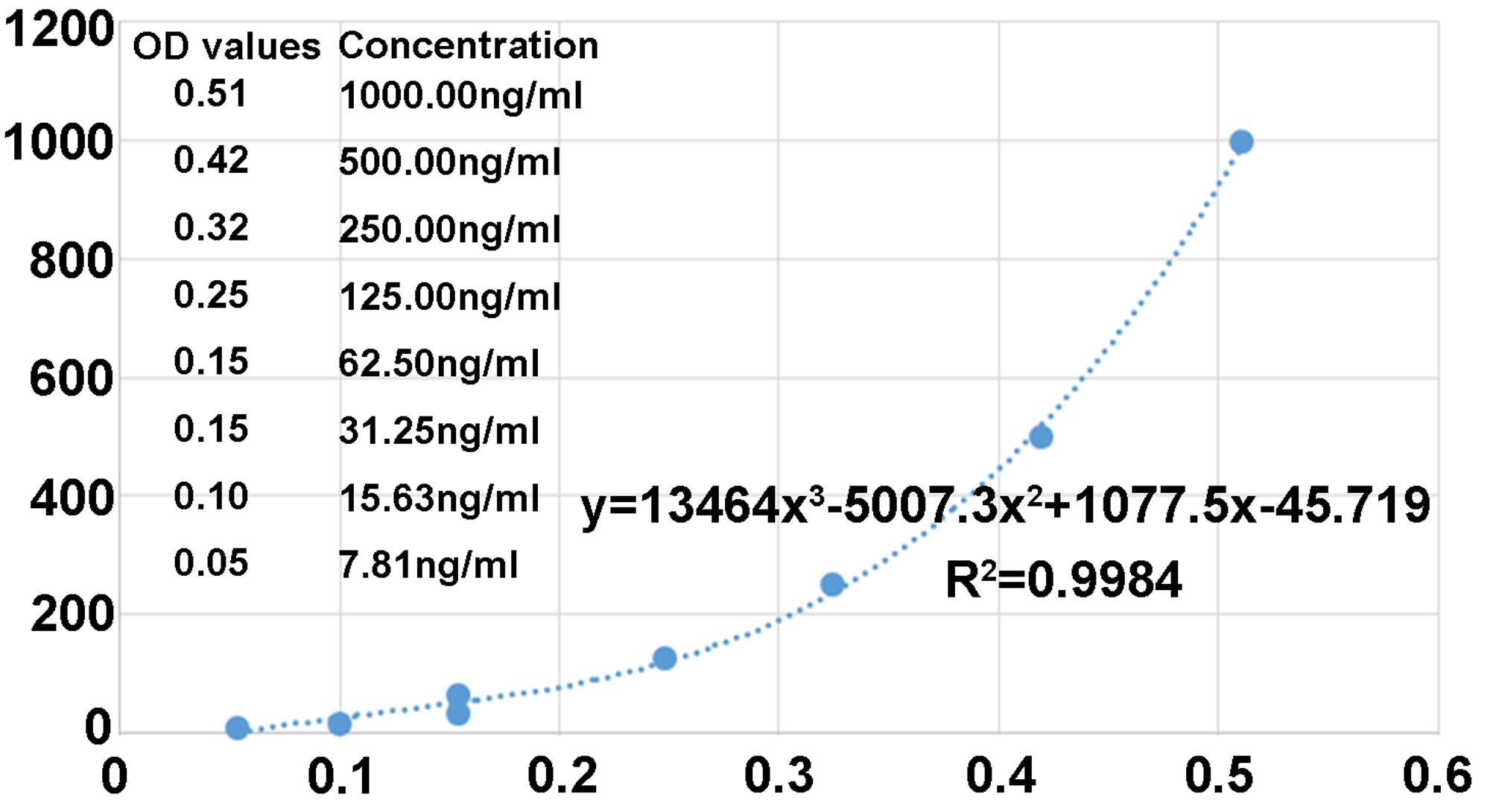

Supplement: Additional file 2: Figure S5. — The standard curve of soluble DC-SIGNR. Human recombinant DC-SIGNR protein was for the standard sample and linear regression was completed successfully, R 2 = 0.9984. The OD value and corresponding concentration of sDC-SIGNR were listed. (TIF 394 kb) [file 13045_2016_383_MOESM2_ESM.tif]

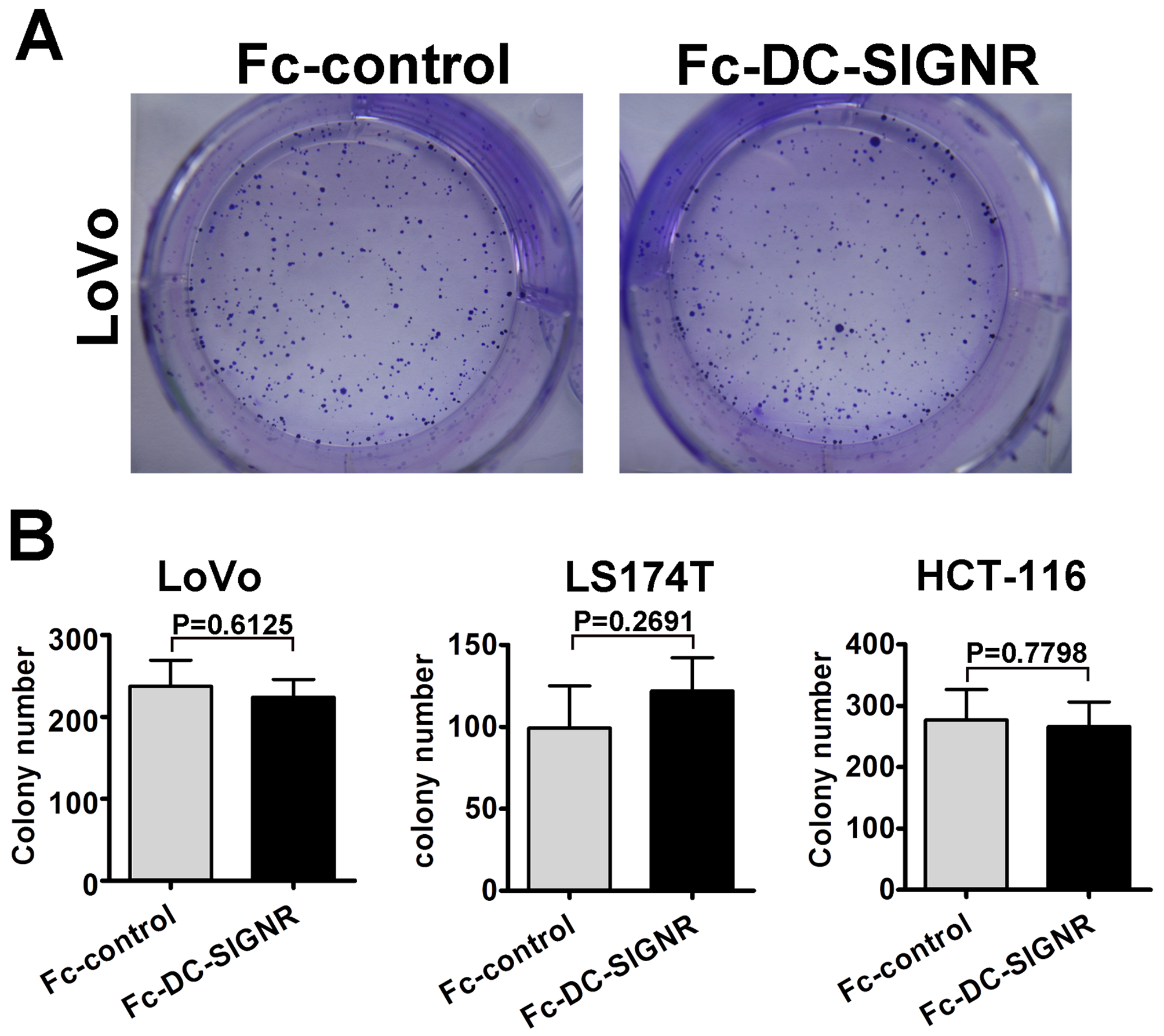

Supplement: Additional file 4: Figure S1. — DC-SIGNR did not promote colony formation in colon cancer cells. (A) The photographs indicated no influence of DC-SIGNR protein on cell proliferation efficiency of LoVo cells. (B) The histograms showed the clone cells number in respective cells. (TIF 1926 kb) [file 13045_2016_383_MOESM4_ESM.tif]

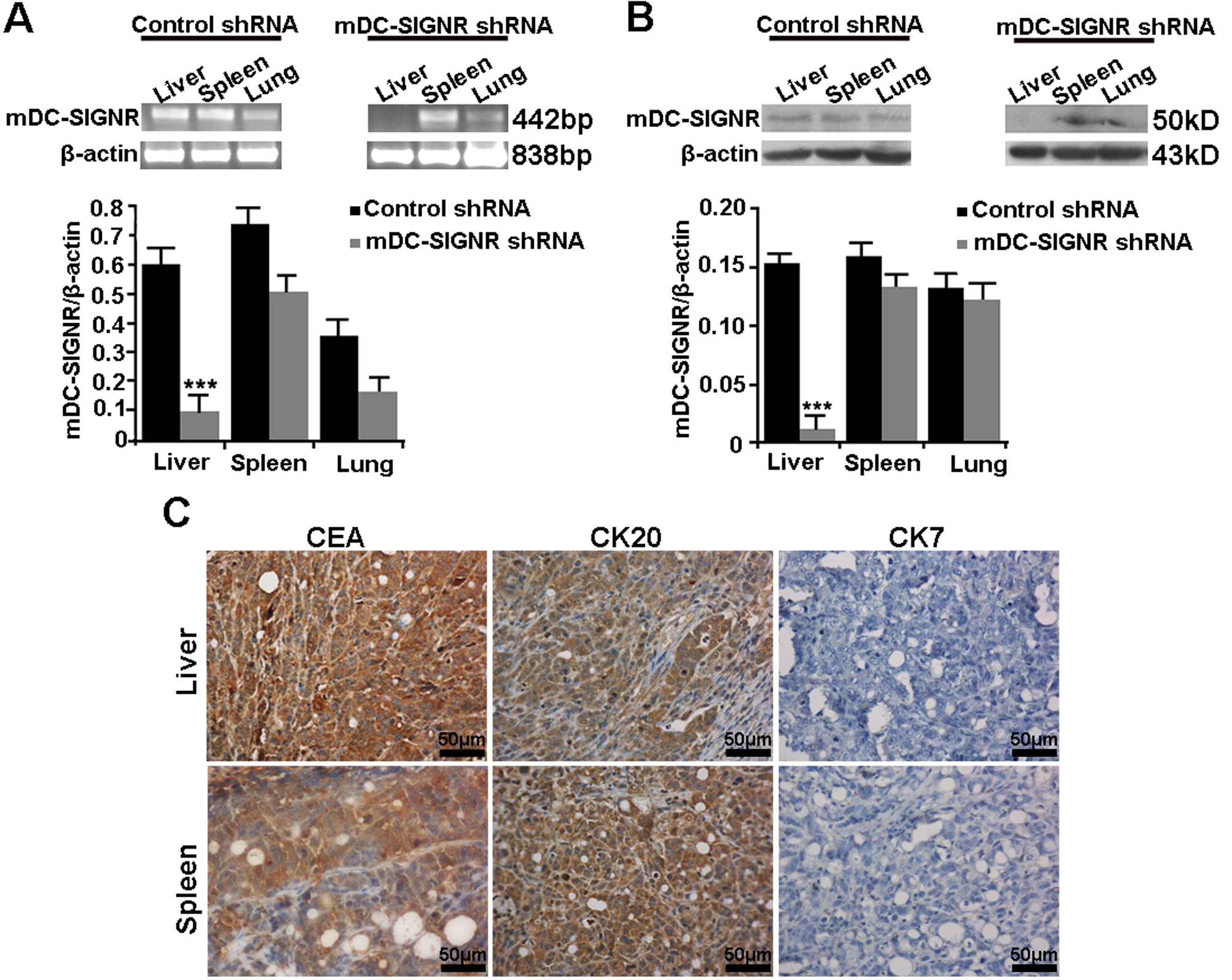

Supplement: Additional file 5: Figure S2. — Mouse DC-SIGNR expression in different organs and the tumor cells matched with colon cancer cells. (A-B) Mouse DC-SIGNR expression in different organs via mouse DC-SIGNR shRNA and control shRNA plasmid injection were detected by RT-PCR and Western Blot. These results showed that the method of hydrodynamic injection of mouse DC-SIGNR shRNA plasmid can significantly suppress mouse DC-SIGNR expression in the liver. However, there was no obvious effect on the mouse DC-SIGNR expression in spleen and lung. (C) Mice were injected with LoVo cells into their spleens after human DC-SIGNR plasmid tail vein injection. The liver and spleen tumor tissues were stained with colon cancer markers CEA, CK20, and CK7. The tumors were positive for CEA and CK20, and negative for CK7, which is a pattern exclusively in colon cancer. ***P < 0.001. (TIF 2552 kb) [file 13045_2016_383_MOESM5_ESM.tif]

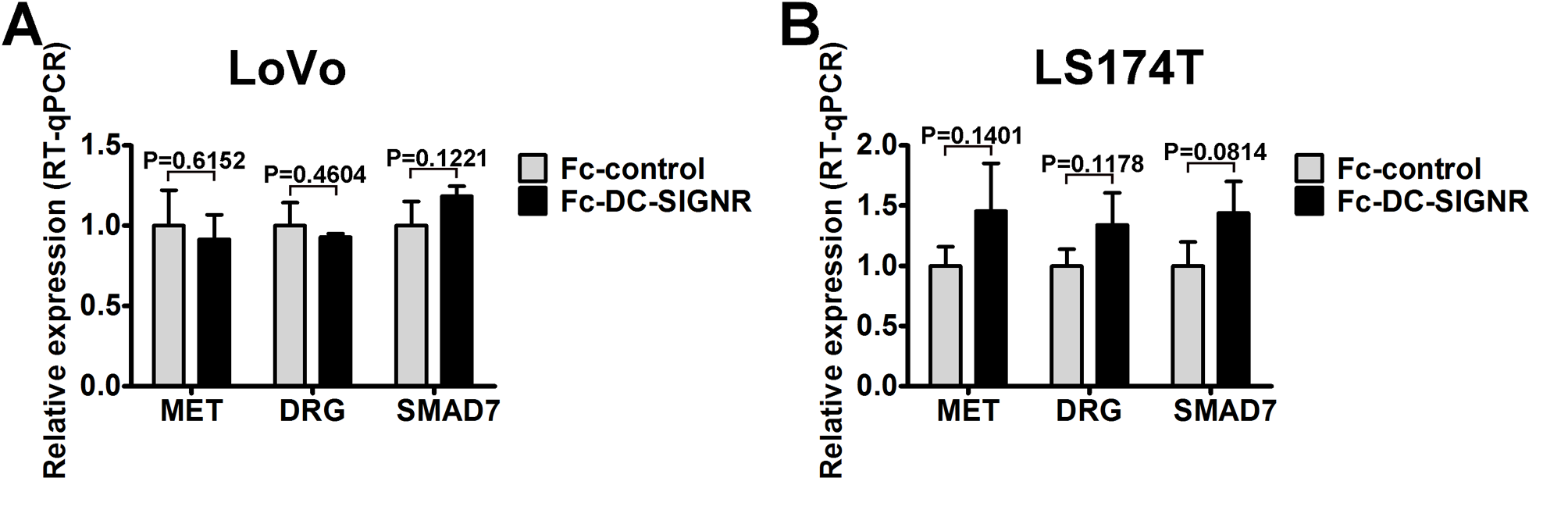

Supplement: Additional file 7: Figure S3. — DC-SIGNR could not regulate the expressions of MET, DRG1 and SMAD7. (A-B) Colon cancer cells LoVo and LS174T were treated with DC-SIGNR protein or control IgG for 4 h. No differences in the expressions of MET, DRG1 and SMAD7 were detected by quantitative real-time PCR. (TIF 217 kb) [file 13045_2016_383_MOESM7_ESM.tif]

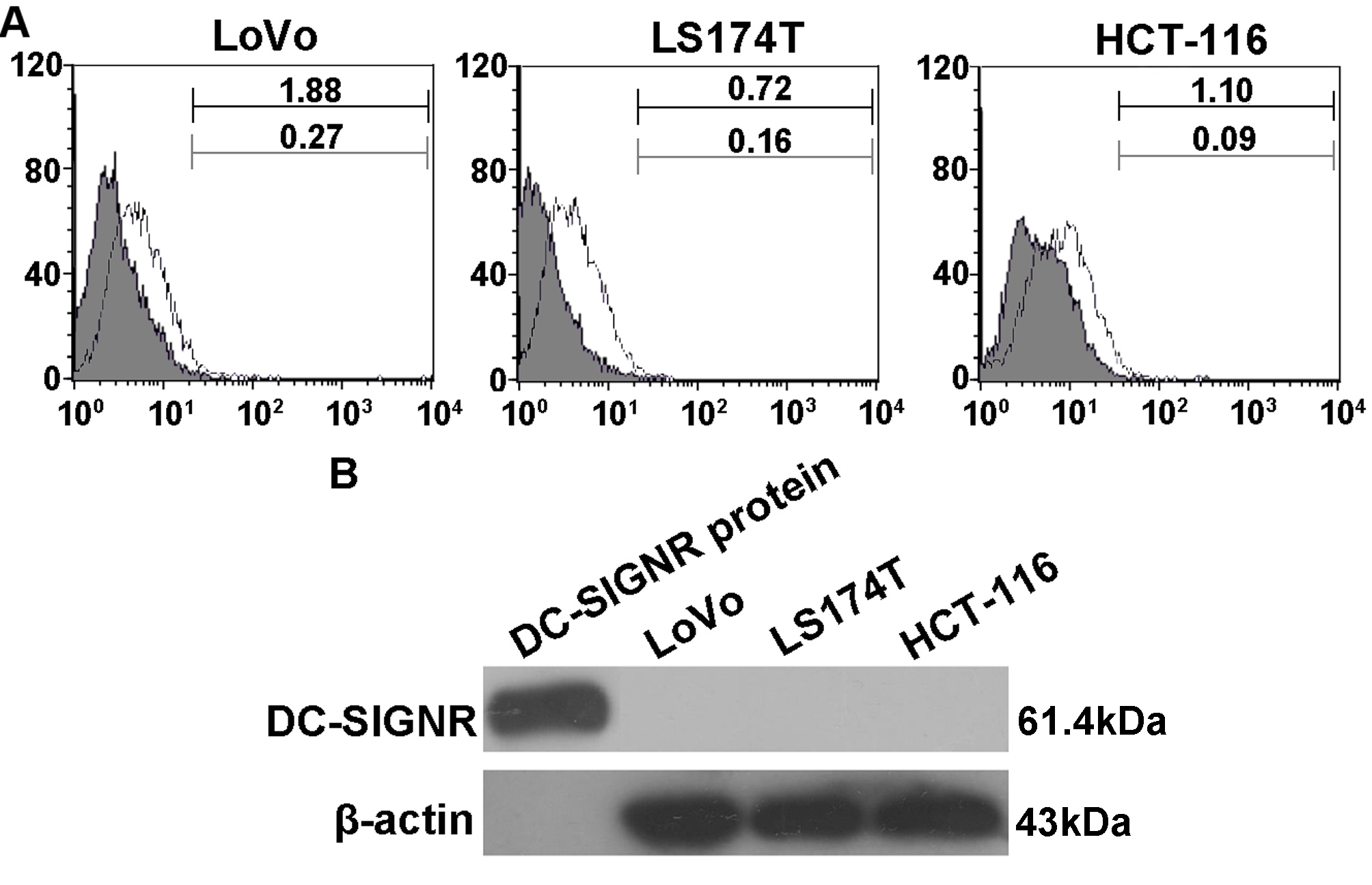

Supplement: Additional file 8: Figure S4. — DC-SIGNR could not be expressed in colon cancer cell lines. (A) The expression of DC-SIGNR was detected by flow cytometry. Results are presented as the percentage of three colon cancer cells expressing DC-SIGNR. (B) Western Blot was used to test the expression of DC-SIGNR in colon cancer cells. Recombinant DC-SIGNR protein was used as positive control. (TIF 463 kb) [file 13045_2016_383_MOESM8_ESM.tif]
