# Supplementary material for: Novel roles of DC-SIGNR in colon cancer cell adhesion, migration, invasion, and liver metastasis
Source: J Hematol Oncol. 2017 Jan 21;10:28. doi: 10.1186/s13045-016-0383-x (PMC5251210; doi:10.1186/s13045-016-0383-x)
Supplement: Additional file 3: Table S3. — The table exhibits the primer sequences used for quantitative real-time PCR. (DOCX 13 kb) [file 13045_2016_383_MOESM3_ESM.docx]

**Supplementary Table3** The primer sequences used for quantitative real-time PCR

| Genes | Forward primer(5’-3’) | Reverse primer(5’-3’) | Product  (bp) |
| --- | --- | --- | --- |
| MT1M | CTAGCAGTCGCTCCATTTATCG | CAGCTGCAGTTCTCCAACGT | 229 |
| MT1B | CAAATGGATCCCAACTGCTC | GCAGCAGCACTTCTTGCAG | 105 |
| MT1G | TCCTGTGCCGCTGGTGTCTC | ACGGGTCACTCTATTTGTACTTGGG | 214 |
| MT1H | GCAAGTGCAAAAAGTGCAAAT | CACTTCTCTGACGCCCCTTT | 115 |
| MT1X | AAGGGACGTCAGACAAGTGC | AAAGATGTAGCAAACGGGTCA | 139 |
| MT1F | CACTGCTTCTTCGCTTCTCTCTT | GCAGGAGCAGCAGCTCTTCT | 159 |
| MMP9 | CCCCTTCACTTTCCTGGGTAA | CGCCACGAGGAACAAACTGT | 151 |
| GAPDH | CCTCAAGATCATCAGCAAT | CCATCCACAGTCTTCTGGGT | 141 |
